# Evolutionary analysis reveals the origin of sodium coupling in glutamate transporters

**DOI:** 10.1038/s41594-025-01652-z

**Published:** 2025-08-25

**Authors:** Krishna D. Reddy, Burha Rasool, Farideh Badichi Akher, Nemanja Kutlešić, Swati Pant, Olga Boudker

**Affiliations:** 1 Department of Physiology and Biophysics, Weill Cornell Medical College, New York, NY, USA.; 2 Department of Molecular Medicine, University of South Florida, Tampa, FL, USA.; 3 Department of Biochemistry, Weill Cornell Medical College, New York, NY, USA.; 4 Howard Hughes Medical Institute, Weill Cornell Medical College, New York, NY, USA.; 5 Present address: Department of Pharmacology, Yale University, New Haven, CT, USA.; 6 Present address: Cold Spring Harbor Laboratory Cancer Center, Cold Spring Harbor Laboratory, Cold Spring Harbor, NY, USA.

## Abstract

Secondary active membrane transporters harness the energy of ion gradients to concentrate their substrates. Homologous transporters evolved to couple transport to different ions in response to changing environments and needs. The bases of such diversification and, thus, principles of ion coupling are unexplored. Here, using phylogenetics and ancestral protein reconstruction, we investigated sodium-coupled transport in prokaryotic glutamate transporters, a mechanism ubiquitous across life domains and critical to neurotransmitter recycling in humans by excitatory amino acid transporters from the solute carrier 1 family. By inferring ancestral prokaryotic transporter sequences during a change in the ion-coupling mechanism, we found an evolutionary transition from sodium-dependent to independent substrate binding and transport. Structural and functional experiments on ancestral transporters suggest that the transition involved allosteric mutations, rendering sodium binding dispensable without affecting the ion-binding sites. Allosteric tuning of transporters’ energy landscapes might be a widespread route of their functional diversification.

Transport of solutes across cell membranes against their concentration gradients is critical for cellular homeostasis. Secondary active membrane transporters catalyze this energetically unfavorable process using the energy of ions flowing down their electrochemical gradients. The ion-binding sites of many transporters are well characterized. However, the three-dimensional (3D) structural properties of transporters necessary to couple substrate and ion movements, and thereby how transporters harvest electrochemical energy, remain elusive.

In the dicarboxylate/amino acid:cation symporter family, the Na^+^-coupling mechanism persisted through millions of years, conserved between archaea and humans; mammalian excitatory amino acid transporters (EAATs; belonging to the solute carrier 1 (SLC1) family) use Na^+^ gradients to clear the neurotransmitter glutamate from the synaptic cleft. Many transporter families, including glutamate transporters, show functional diversification using Na^+^ or H^+^ gradients (Fig. 1a)^1,2^. The cell’s environment and needs provide the selective pressure to use specific ions such that when cell membranes are H^+^-leaky or Na^+^ is abundant, Na^+^-coupled transport predominates. In contrast, in environments where Na^+^ is scarce, such as in fresh water, certain soils and fermented foods, Na^+^ coupling is no longer beneficial and is eliminated. We used phylogenetic analysis, ancestral protein reconstruction (APR), and structure-function characterization of inferred ancestral proteins to understand how prokaryotic glutamate transporters transitioned away from Na^+^ coupling. Contrary to our expectations, the transition did not involve promiscuous ion coupling^3,4^ or a gradual loss of Na^+^-binding sites. Instead, allosteric changes first made Na^+^ binding dispensable to function in a distinct evolutionary step, and only then did the transporters lose their Na^+^-binding residues.

Energetically linked substrate and Na^+^ binding underlies symport in Na^+^-coupled glutamate transporter homologs, including archaeal Glt_Ph_ and Glt_Tk_. A higher extracellular Na^+^ concentration ensures a greater probability of substrate binding from the outside and release on the inside of the membrane, providing directionality to the transport cycle and leading to concentrative uptake^5–12^. The binding sites for the substrate and three symported Na^+^ ions are located within a dynamic transport domain, which moves between extracellular and intracellular positions (outward-facing and inward-facing states, Extended Data Fig. 1) in an elevator-like motion relative to a stationary scaffold and trimerization domain^13–22^. The transport mechanism and homotrimeric architecture are conserved across glutamate transporters^23–27^. Na^+^ binding to two sites (Na1 and Na3) induces the formation of a high-affinity substrate-binding pocket, resulting in substrate and the third Na^+^ (Na2) binding and closure of the helical hairpin 2 (HP2) gate, which precedes translocation^8,9,28–32^ (Fig. 1b,c). Thus, ion-coupled binding and transport occur because the high-affinity substrate-binding state is a high-energy state only accessible as Na^+^ binds. Accordingly, mutations impacting all three Na^+^-binding sites diminish Na^+^-coupled substrate binding and transport^3,10,33–36^.

Using APR, we reconstructed and characterized ancestral transporter sequences (Fig. 1d); we found that an intermediate ancestral transporter (Anc^Int^) between Na^+^-coupled and H^+^-coupled ancestral transporters showed Na^+^-independent substrate binding and transport, despite preserved Na^+^-binding sites. Cryo-electron microscopy (cryo-EM) structures showed that Anc^Int^ accessed the high-affinity substrate-binding state spontaneously, suggesting that its energy is lower than in Na^+^-coupled transporters and explaining why Na^+^ binding was no longer necessary. Detailed evolutionary analysis revealed that the shifted energetic balance from the low-affinity to the high-affinity states was because of allosteric changes distant from the ion-binding and substrate-binding sites, and mutations impacting just two residues in Anc^Int^ restored Na^+^ coupling. We propose that allosterically changing the relative energy of functional states in transporters constitutes an evolutionary mechanism allowing the diversification of strictly specific ion coupling observed in present-day transporters. Our work reveals key principles underlying Na^+^ coupling, showcases the feasibility and power of structure–function APR in untangling the complex mechanisms of membrane transporters, and suggests a general evolutionary mechanism underlying adaptations to different ionic environments in secondary active transporters.

## Results

### An evolutionary intermediate between Na^+^-coupled and H^+^-coupled transporters

**Fig. 1 |:**
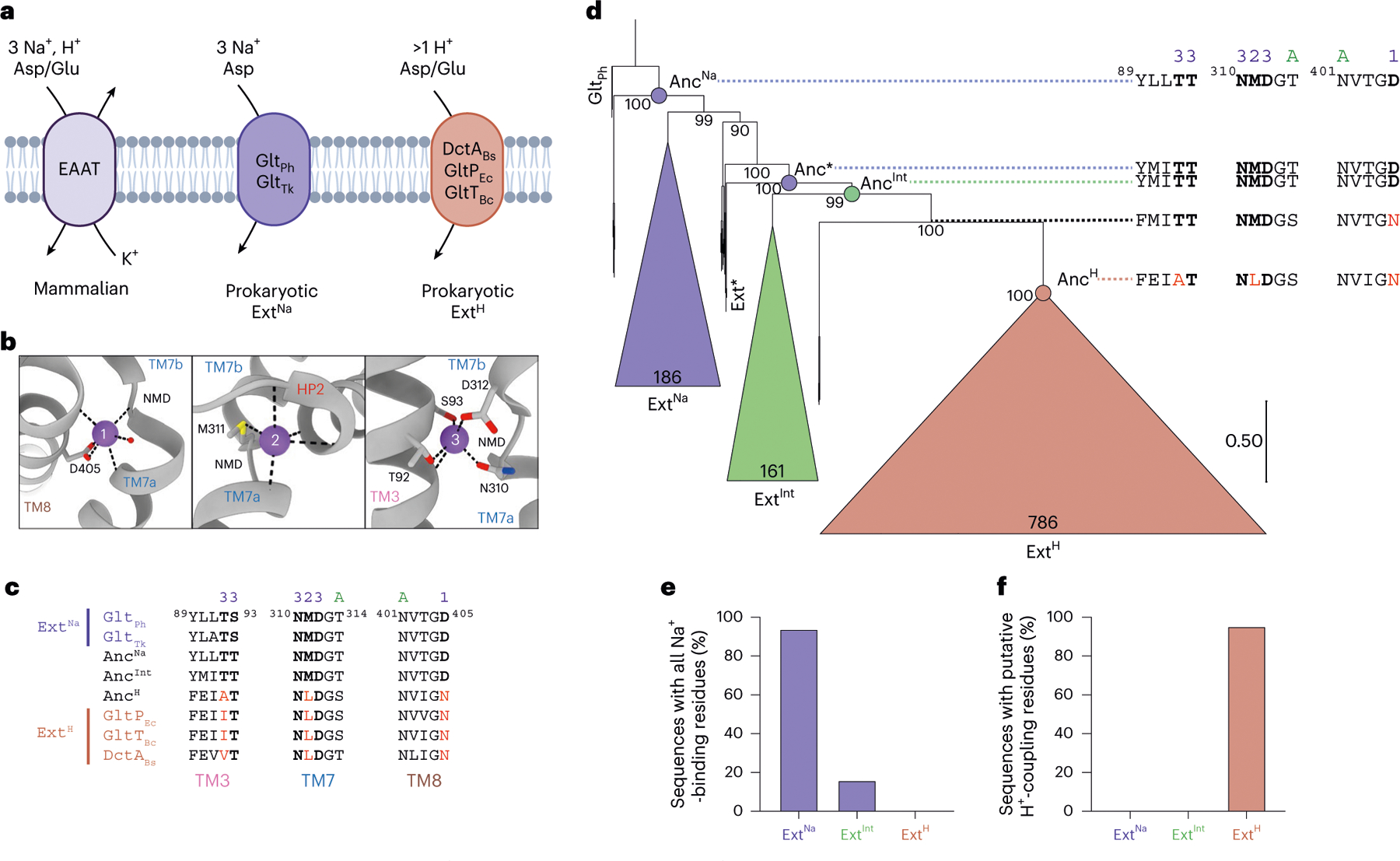
Mapping the evolutionary transition from sodium to proton coupling. **a**, Ion-coupling stoichiometries of characterized concentrative transporters in the glutamate transporter family. **b**, The architecture of Na^+^-binding sites in Glt_Ph_ (PDB 6X15). Bound Na1–Na3 are shown as purple spheres. Dotted lines emphasize the coordinating interactions with sidechains, shown as sticks, and main chain carbonyl oxygens, shown as cartoons. **c**, Sequence alignment of experimentally characterized prokaryotic homologs and inferred ancestral transporters from **d**. The numbers above the alignment refer to the Na^+^-binding sites and ‘A’ refers to side chains coordinating the substrate l-aspartate. Signature sequence changes associated with the transition to H^+^ coupling are shown in red. **d**, Reduced ML phylogeny of glutamate transporters in Firmicutes. The collapsed Ext^Na^ (purple), Ext^Int^ (green) and Ext^H^ (orange) clades are proportional to the number of the comprising sequences, which is shown within the clade. Numbers at nodes represent corresponding UFBoot2 values. Nodes corresponding to the reconstructed ancestral sequences are highlighted in the corresponding color and labeled Anc^Na^, Anc*, Anc^Int^ and Anc^H^. **e**, Percentage of sequences in each clade containing all Na^+^-binding site residues ([S/T]92–93, N310, [M/S]311, [D/E]312 and [D/E]405). **f**, Percentage of sequences in each clade containing signature H^+^-coupling residues ([A/L/I/V/M/F]92, L311 and N405).

We used phylogenetic analysis and APR to understand how bacterial glutamate transporters might have changed their ion-coupling preference (Extended Data Fig. 2). The approach can reveal minimal subsets of stepwise, chronologically ordered changes required to alter functions, including epistatic changes, which are insufficient for the functional switch but are prerequisites for it^37^. Subsequent inference of ancestral sequences along an evolutionary pathway provides a platform to test evolutionary and mechanistic hypotheses^38–44^. We selected sequences in the Firmicutes/Bacillota phylum to recapitulate the transition from Na^+^ to H^+^ coupling, rooting the phylogenetic tree to archaeal Na^+^-coupled Glt_Ph_ (Methods and Extended Data Fig. 3a–d). We identified three major monophyletic clades in our tree (Fig. 1d), with two predictably displaying conserved signature sequences of Na^+^ and H^+^ coupling (Fig. 1e,f and Extended Data Fig. 3d). We refer to these present-day extant sequences as Ext^Na^ and Ext^H^ clades. Strikingly, we also observed an intermediate clade Ext^Int^ between Ext^Na^ and Ext^H^ clades. Only 16% of its sequences contain all Na^+^-coordinating residues, and none contain all H^+^-coupling signature residues (Fig. 1e,f and Extended Data Fig. 3d). This branch was robust to perturbation and reconstructed with high confidence on the basis of a UFBoot2 bootstrap value > 95% (Fig. 1d)^45^. The extant sequences closest to Ext^Int^ with the full complement of Na^+^-coordinating residues are referred to as Ext*.

**Fig. 2 |:**
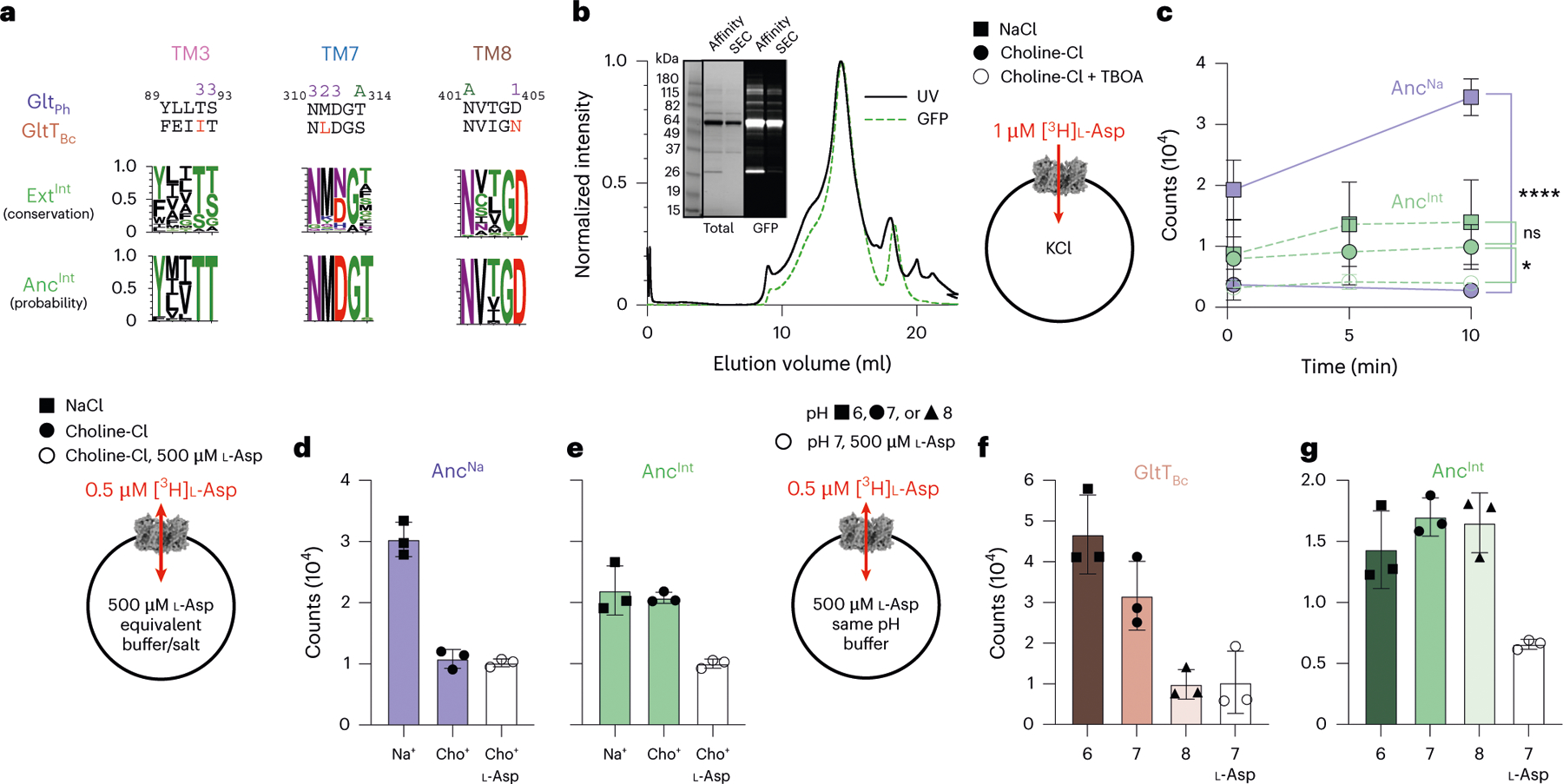
Substrate transport by Anc^Int^ is independent of Na^+^ and H^+^. **a**, Comparison of the signature Na^+^-coupling residues in present-day Ext^Int^ transporters and inferred Anc^Int^. Ext^Int^ WebLogo represents the conservation of transporter residues in the Ext^Int^ clade. Anc^Int^ WebLogo is a visual representation of the PP per site in the ancestral reconstruction; the topmost residue at each site represents the ML residue. **b**, Representative SEC profiles of GFP-tagged Anc^Int^ purified in ligand-free conditions. Black and dotted green lines correspond to the relative optical absorption at 280 nm and GFP fluorescence (excitation, 485 nm; emission, 510 nm), respectively. Inset, SDS–PAGE analysis of GFP-tagged Anc^Int^, stained with total protein Coomassie stain (total) or imaged by in-gel GFP fluorescence (GFP). ‘Affinity’ refers to elution following streptactin affinity purification. ‘SEC’ refers to peak fractions eluting at 14.5 ml following SEC. **c**, [^3^H]L-aspartate uptake in the presence (filled squares) or absence (filled circles) of a Na^+^ gradient. Left, simplified proteoliposome reaction setup. All experiments were performed at an estimated potential of −102 mV, generated by valinomycin-mediated K^+^ efflux. Lines represent Anc^Na^ (solid purple) or Anc^Int^ (dashed green). Open circles represent Anc^Int^ without a Na^+^ gradient and in the presence of 1 mM dl-TBOA. Statistical significance at 10 min was measured using unpaired two-tailed *t*-tests. ****P < 0.0001 and *P = 0.0193; not significant (NS), *P* = 0.3431. Error bars are the means ± s.d. of three (Anc^Na^) or four (Anc^Int^) independent experiments, each the average of at least two technical replicates; error bars not shown are smaller than the displayed symbol. **d**–**g**, Exchange activity of ancestral transporters. All experimental setups had external 0.5 μM [^3^H]L-aspartate and internal 500 μM unlabeled L-aspartate; negative controls (clear circles) contained an additional external 500 μM unlabeled L-aspartate. Error bars are the means ± s.d. of three independent experiments, each the average of at least two technical replicates. **d**,**e**, Sodium-dependent counterflow of Anc^Na^ (**d**; purple) or Anc^Int^ (**e**; green) in the presence of internal and external 10 mM NaCl (filled squares) or choline chloride (filled circles). **f**,**g**, pH-dependent counterflow of GltT_Bc_ (**f**; orange) or Anc^Int^ (**g**; green) in the presence of internal and external KH_2_PO_4_ and K_2_HPO_4_ at pH 6 (filled squares), 7 (filled circles) or 8 (filled triangles).

Next, we inferred ancestral sequences by determining posterior probabilities (PPs) of each amino acid occurring at each position (site) in the multiple-sequence alignment (Extended Data Fig. 4a–f). Each node’s final maximum likelihood (ML) amino acid sequence is constructed using the most probable amino acid at every site. We reconstructed sequences corresponding to the closest ancestor to the Na^+^-coupled root (Anc^Na^) and the ancestor of the intermediate Ext^Int^ clade (Anc^Int^); the average PPs per site were 0.87 and 0.85, respectively (Extended Data Fig. 4a,b), which is sufficiently high for APR studies^46,47^. As expected, reconstructed Anc^Na^ sequences showed signature sequences of Na^+^-coupled transporters (Fig. 1c and Extended Data Figs. 4a and 5). Surprisingly, Anc^Int^ also featured the full complement of Na^+^-binding residues with high PPs, contrasting with their poor conservation among the descendent present-day transporters (Figs. 1c and 2a and Extended Data Figs. 4a and 5). These observations suggest a lack of selective pressure on Na^+^-binding residues in Anc^Int^, raising the question of its functional properties.

To design ancestral constructs for protein expression, we replaced poorly aligned highly variable loops and tails, excluded from the APR analysis, with those from an Ext* sequence with the highest identity to Anc^Int^ (Extended Data Fig. 5). To minimize the impact of these regions on our comparisons between ancestors, we used the same loops and tails for all ancestral constructs. Most of these were single-residue insertions, except for the C-terminus and the loop between transmembrane helix 3 (TM3) and TM4. Although these insertions might affect protein stability, flexibility and function, previous studies in Glt_Ph_ suggest that these regions are not directly involved in ion coupling. The C-terminus has been deleted in structural and functional studies^13^, and cleavage of the 3–4 loop affected transport kinetics but not ion coupling^48^. We expressed ancestors in *Escherichia coli* with or without a C-terminal optimized GFP^49^ and purified them (Fig. 2b and Supplementary Fig. 1a–c). While there are several bands above and below the primary band, they correlate with GFP fluorescence in gels and size-exclusion profiles. Therefore, these are likely misfolded or aggregated species. Despite extensive optimization, the ancestral proteins expressed ~10-fold lower than extant prokaryotic homologs, yielding ~20–100 μg of purified proteins per L of culture.

### The ancestral intermediate transporter is uncoupled from Na^+^

When we purified and reconstituted Anc^Na^ into proteoliposomes, we observed a time-dependent accumulation of [^3^H]l-aspartate in vesicles in the presence of an inward Na^+^-gradient. In contrast, no [^3^H]l-aspartate was retained in the absence of Na^+^ (Fig. 2c). These results suggest that Anc^Na^ is adequately folded and recapitulates expected Na^+^ coupling. When we repeated the experiment with Anc^Int^, we observed lower [^3^H]l-aspartate retention unaffected by the removal of Na^+^ but reduced in the presence of the competitive inhibitor dl-TBOA (Fig. 2c), suggesting that Anc^Int^ in proteoliposomes might be able to bind and transport l-aspartate independently of Na^+^. To demonstrate this, we measured [^3^H]l-aspartate accumulation in Anc^Int^ proteoliposomes under exchange conditions^1,50^, with 500 μM unlabeled l-aspartate inside the vesicles and 0.5 μM [^3^H]l-aspartate outside. Anc^Int^ accumulated substrate independently of Na^+^ or pH, unlike the respective controls, Anc^Na^ and GltT_Bc_ (Fig. 2d–g). Together, this suggests that Anc^Int^ functions as a cation-independent exchanger.

**Fig. 3 |:**
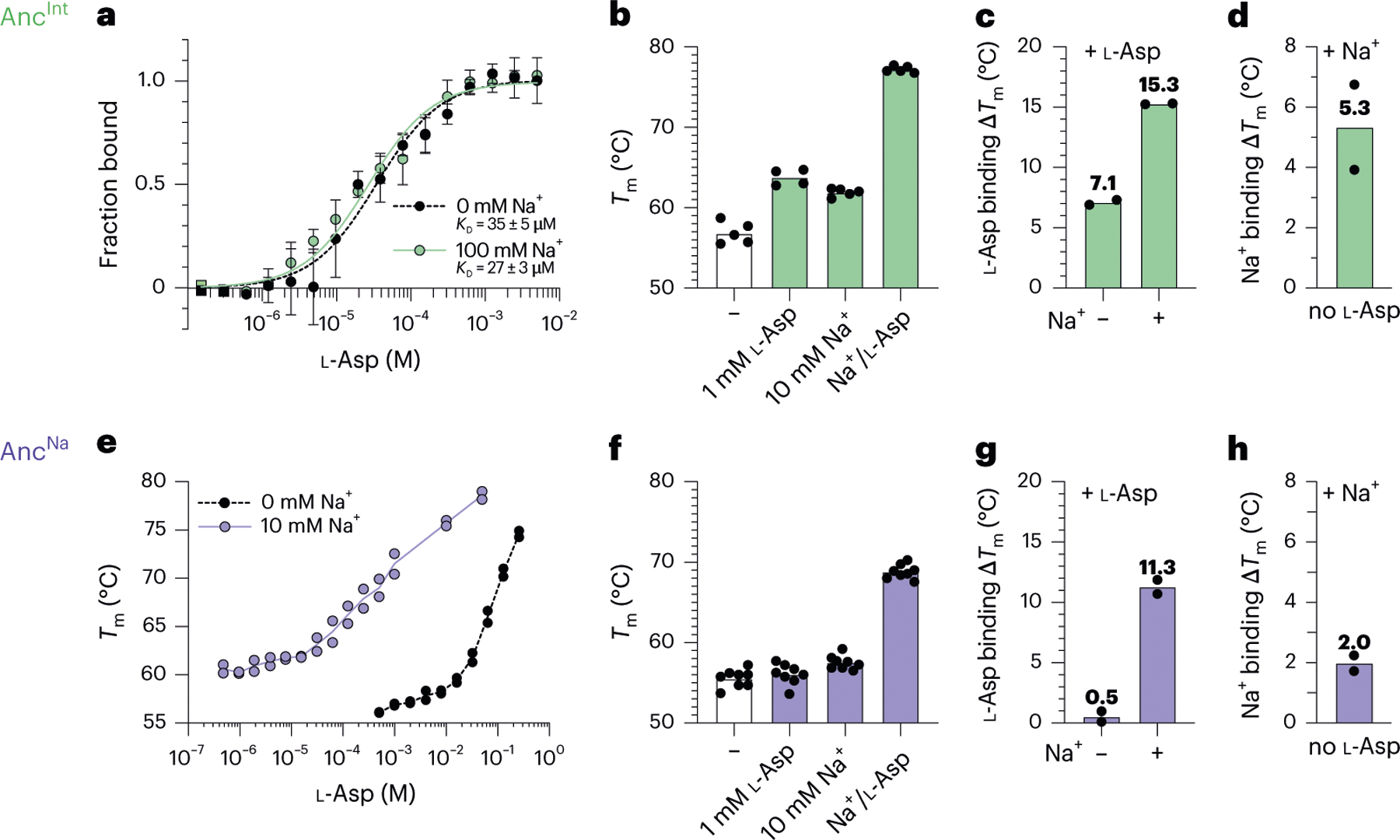
Anc^Int^ binds substrate independently of Na^+^. **a**, l-Aspartate binding by Anc^Int^ in 100 mM NaCl (green circles) or 100 mM choline chloride (black circles), measured by MST. The data shown are the means ± s.d. from three independent experiments; however, the first two points (squares) had points excluded because of aggregation and, therefore, do not have error bars. The best-fit *K*_D_ values and s.e.m. in NaCl and choline chloride were 27 ± 3 μM and 35 ± 5 μM, respectively. **b**, Anc^Int^ *T*_m_ in buffer alone (ligand-free), 1 mM l-aspartate, 10 mM NaCl or both NaCl and l-aspartate. The data shown are from two independent experiments, each with several technical replicates. **c**,**d**, Anc^Int^ Δ*T*_m_ values upon addition of l-aspartate in the presence and absence of Na^+^ (**c**) and in the presence of Na^+^ but absence of l-aspartate (**d**). **e**, *T*_m_ of Anc^Na^ as a function of l-aspartate concentration in either 0 mM NaCl (black circles) or 10 mM NaCl (purple circles). Lines are to guide the eye and represent the means of the two displayed independent replicates. **f**, Anc^Na^ *T*_m_ in buffer alone (ligand-free), 1 mM l-aspartate, 10 mM NaCl or both NaCl and l-aspartate. The data shown are from two independent experiments, each with several technical replicates. **g**,**h**, Anc^Na^ Δ*T*_m_ values upon the addition of l-aspartate in the presence and absence of Na^+^ (**g**) and in the presence of Na^+^ but absence of l-aspartate (**h**); data are from two independent experiments.

We used microscale thermophoresis (MST) to measure substrate-binding affinity to Anc^Int^. Anc^Int^ binds l-aspartate with similar micromolar affinities in the absence and presence of 100 mM Na^+^ (Fig. 3a). Notably, the MST signal-to-noise values upon l-aspartate binding ranged from 8.6 to 19.5, reflecting the high confidence of the binding isotherms (Supplementary Fig. 2a). We confirmed this finding using nano-differential scanning fluorimetry (nanoDSF) to measure ligand-dependent protein melting temperatures (*T*_m_) as a qualitative proxy for ligand binding^51,52^. Adding l-aspartate to Anc^Int^ in the absence of Na^+^ increased *T*_m_ by 7.1 °C, suggesting that, as expected, l-aspartate can bind without Na^+^ (Fig. 3b,c and Supplementary Fig. 2c). Notably, 10 mM Na^+^ also stabilized the protein by 5.3 °C without l-aspartate and by 13.5 °C in the presence of 1 mM l-aspartate, suggesting that the ions can still bind to the transporter regardless of substrate binding (Fig. 3c,d and Supplementary Fig. 2c). In Anc^Na^, the MST signal-to-noise ratio was insufficient to accurately determine binding affinities, likely because of changes in measurable parameters, including charge, hydration shell or conformation^53^. In nanoDSF l-aspartate titrations, much lower concentrations of l-aspartate were required to observe substrate binding in 10 mM Na^+^ as compared to 0 mM Na^+^ (Fig. 3e). We observed insignificant or small *T*_m_ increases when adding 1 mM l-aspartate or 10 mM Na^+^ separately compared to the ligand-free Anc^Na^ (Fig. 3f,h and Supplementary Fig. 2b). In contrast, we observed a dramatic 11.3 °C increase when adding 1 mM l-aspartate in the presence of 10 mM Na^+^(Fig. 3f,g). Thus, l-aspartate and Na^+^ bind to Anc^Na^ cooperatively, as reported for Na^+^-coupled transporters^1,8,10,11,12,54,55^.

These results suggest that, unlike in Na^+^-coupled transporters, l-aspartate and Na^+^ can bind to Anc^Int^ independently; therefore, substrate and ion binding are ‘uncoupled’. Notably, while we did not observe any Na^+^ stimulation in either MST or exchange experiments, the Δ*T*_m_ of l-aspartate was larger in the presence of Na^+^ (15.3 °C versus 7.1 °C), suggesting residual cooperativity. However, thermal melts are not thermodynamic measurements and Δ*T*_m_ values cannot be directly related to ligand affinity. Overall, we conclude that the intermediate Ext^Int^ clade functionally diverged from the Na^+^-coupled Ext^Na^ transporters by uncoupling substrate from Na^+^ binding. We attempted to verify that the reconstructed Anc^Int^ was robust to phylogenetic uncertainty using ‘AltALL’ sequences where all ambiguous residues are varied^46^ but these produced misfolded proteins. Instead, we validated the observed functional divergence by examining the necessary and sufficient sequence changes, as discussed below.

### Anc^Int^ accesses high-affinity states without Na^+^

To visualize how Na^+^-independent aspartate binding occurs, we purified Anc^Int^, reconstituted it into MSP1E3 lipid nanodiscs (Supplementary Fig. 3), exchanged it into a buffer containing no Na^+^ or any other alkali cations (20 mM HEPES pH 7.4 and 100 mM NMDG-Cl), and froze cryo-EM grids after and before adding 1 mM l-aspartate (that is, in bound and ligand-free conditions, respectively) (Supplementary Figs. 4 and 5). As a Na^+^-coupled control, we reconstituted Glt_Ph_ into MSP1E3 lipid nanodiscs and froze grids under ligand-free conditions (Supplementary Fig. 6).

**Fig. 4 |:**
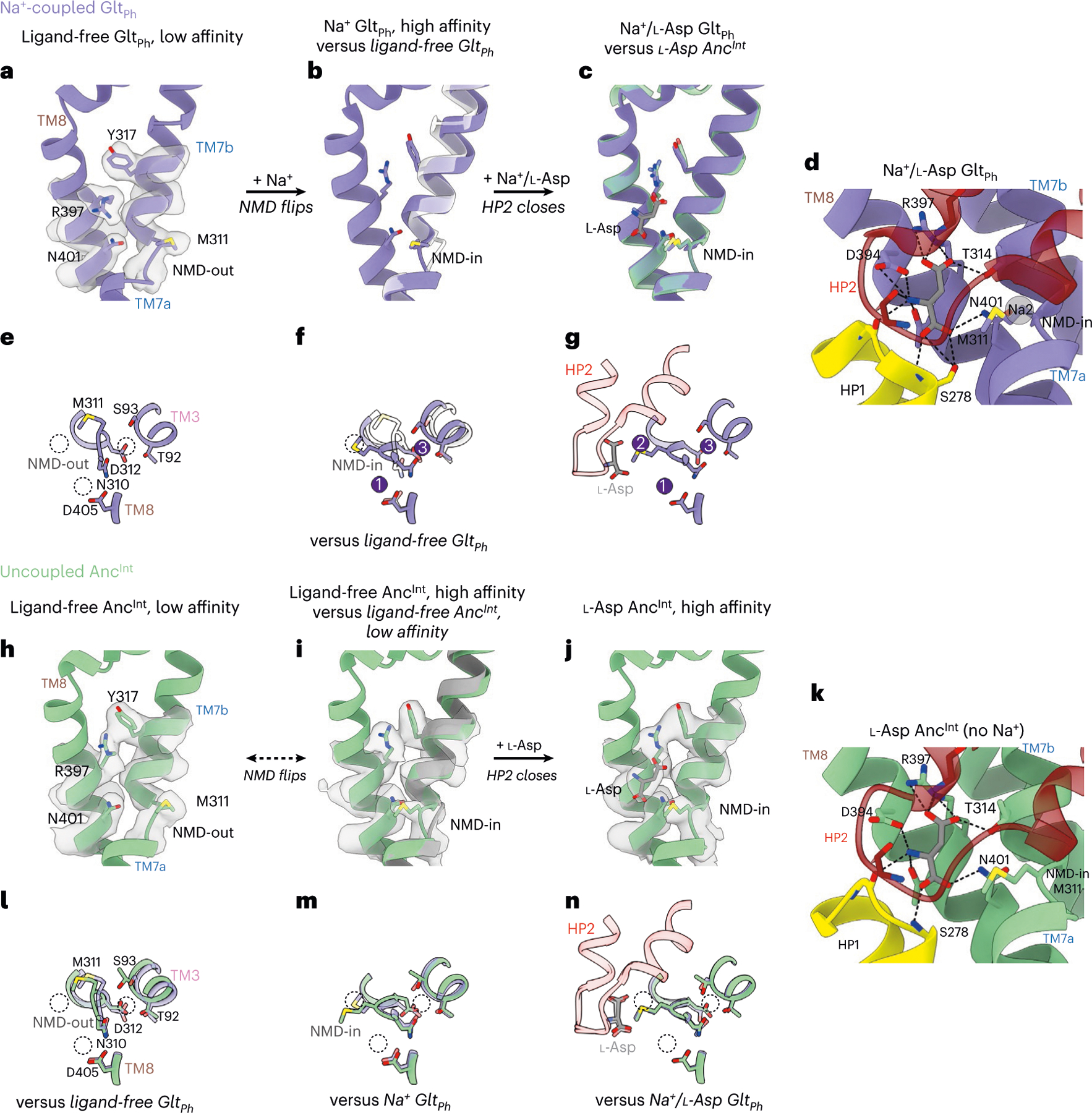
Substrate-binding mechanism in Na^+^-coupled and uncoupled transporters. All numbered residues use Glt_Ph_ numbering. All structures are aligned on the HP1/TM7a region (residues 259–309 in Glt_Ph_). All densities are in gray and are sharpened and contoured at 10σ. Purple spheres represent bound Na^+^ ions, and dashed circles represent unoccupied Na^+^ sites. Na^+^-coupled substrate binding of Glt_Ph_ (purple; **a**–**g**) and uncoupled substrate binding of Anc^Int^ (green; **h**–**n**). Panels depict the substrate-binding (**a**–**d**,**h**–**k**) and ion-binding (**e**–**g**,**l**–**n**) sites. **a**,**e**, In ligand-free Glt_Ph_ (outward-facing), both substrate-binding and ion-binding sites are distorted, forming a low-affinity configuration. **b**,**f**, Superposition of Na^+^-bound Glt_Ph_ (PDB 7AHK) and translucent ligand-free Glt_Ph_. Upon Na1 and Na3 binding, the NMD loop and M311 side chain flip into the pocket. TM3 (S92-T93) moves closer to the NMD loop to form the Na3 and Na1 sites and the M311, R397 and N401 side chains adjust to form Na2-binding and l-aspartate-binding sites. These changes produce the high-affinity substrate-binding state. **c**,**d**,**g**, Na^+^/l-aspartate-bound Glt_Ph_ (PDB 6X15) demonstrates subsequent l-aspartate/Na2 binding and HP2 closure; l-aspartate coordination was determined by hbonds in ChimeraX. **h**,**i**,**l**,**m**, In ligand-free conditions, Anc^Int^ visits both low-affinity (**h**,**l**) and high-affinity (**i**,**m**) states. Spontaneous NMD loop and side-chain movements are similar to those induced by Na^+^ binding in Glt_Ph_ (translucent purple, **l**,**m**). **j**,**k**,**n**, l-Aspartate binding to the preformed site and HP2 closure in Anc^Int^ without Na^+^; l-aspartate coordination is similar to Glt_Ph_.

First, we compared the structures of Glt_Ph_ and Anc^Int^ in ligand-free conditions. Cryo-EM imaging of Glt_Ph_ revealed the presence of two structural classes with final maps at 2.7-Å and 3.1-Å resolution (Extended Data Figs. 6 and 7, Supplementary Fig. 6 and Table 1). A highly conserved nonhelical loop N310-M311-D312 (NMD) motif, which breaks TM7 into TM7a and TM7b, is the nexus for solute binding and participates in coordinating Na1, Na2, Na3 and substrate through its side chain and main chain atoms. In both classes, the NMD motif is in a ‘flipped-out’ configuration, which distorts substrate-binding and Na^+^-binding sites (Fig. 4a,e). We call these conformations, characteristic of ligand-free Na^+^-coupled transporters, ‘low-affinity’ states^9,15,16,30,56^. The two ligand-free Glt_Ph_ structural classes differ in the position of the transport domain. One corresponds to the outward-facing state (Fig. 4a,e) and the other corresponds to a previously observed intermediate^16,21,30,57^ where the transport domain moves toward the inward-facing state by about one quarter of the way (Extended Data Figs. 7 and 8a). The HP2 gate is open in the outward-facing states, propped open by M311, and closed in in the intermediate, with M311 pointing out into the lipid from underneath (Fig. 4a and Extended Data Fig. 8a).

Previously determined Glt_Ph_ and Glt_Tk_ structures show that, upon Na^+^ binding to Na1 and Na3, Na^+^-coupled transporters restructure from the low-affinity ligand-free state to a ‘high-affinity’ state through allosterically coupled changes^8,9,28,10^ (Fig. 4b,f). Their NMD motifs go from the flipped-out to the flipped-in configuration, repositioning M311 proximal to the substrate (Fig. 4b) and altering the tilt of TM7b^30^. R397 moves from occupying the substrate-binding site to forming a cation–*π* interaction with Y317 in TM7b (Fig. 4b), with its guanidium group in place to salt-bridge with l-aspartate. Moreover, N401 repositions slightly to coordinate l-aspartate optimally^30^. Together, these changes underlie high-affinity binding by optimally positioning residues coordinating the substrate and the additional Na2 (Fig. 4c,d,g). Following substrate binding, HP2 closes (Fig. 4g), facilitating translocation of the transport domain to the inward-facing state.

Cryo-EM imaging of ligand-free Anc^Int^ revealed three outward-facing structural classes, all refined to 3.0-Å resolution (Extended Data Fig. 7, Supplementary Fig. 5 and Table 1). One class resembles the low-affinity ligand-free Glt_Ph_ with a flipped-out NMD motif and an empty binding pocket; however, R397_369_ is already in place to coordinate l-aspartate (hereafter, regular text and subscript correspond to Glt_Ph_ and Anc^Int^ numbering, respectively) (Fig. 4h). The Na^+^-binding sites are distorted similarly to ligand-free Na^+^-coupled transporters (Fig. 4l), except that T92_78_ and [T/S]93_79_ in the Na3 site are already correctly positioned.

However, unlike ligand-free Glt_Ph_, we also observed two ligand-free Anc^Int^ classes in high-affinity states, with substrate-binding and Na^+^-binding site architectures like those of Glt_Ph_ after Na^+^ binding to Na1 and Na3 sites (Fig. 4i,m). The NMD motifs are flipped in, R397_369_ and N401_373_ are correctly oriented for l-aspartate coordination (Fig. 4i), and all residues in Na1 and Na3 sites are configured like in Na^+^-bound Glt_Ph_ (Fig. 4m). Water molecules likely occupy the sites in the absence of Na^+^ but the resolution is insufficient to model them, and computational studies will be needed to understand how they compensate for the missing ions. One of the classes shows an HP2 structurally identical to substrate-bound Anc^Int^, except with an unresolved tip (the loop connecting the two helical arms of the hairpin) and an empty substrate-binding pocket (Extended Data Fig. 8d). In the other class, an ambiguous density occupies the pocket that is too large for l-aspartate and might correspond to a spuriously bound buffer component; this site might be generally attractive to anions^28^ (Extended Data Fig. 8b). We excluded this structure and inward-facing classes from further consideration; the latter are poorly resolved and have notable regions of missing density in the transport domain (Extended Data Fig. 7). We expect similar structures of the substrate-binding and ion-binding sites in the inward-facing and outward-facing states on the basis of observations in other glutamate transporter family members^15,16,30^.

### Binding sites are unaltered in uncoupled transporters

Imaging of aspartate-bound Anc^Int^ in Na^+^-free conditions revealed a single outward-facing structural class, which we refined to 3.4-Å overall resolution (Extended Data Figs. 6 and 7, Supplementary Fig. 4 and Table 1). The only structural differences between aspartate-bound Anc^Int^ and high-affinity ligand-free Anc^Int^ are that its HP2 tip is well structured and closed, and the substrate-binding pocket is occupied by a strong nonprotein density, which we modeled as l-aspartate (Fig. 4j,k,n and Extended Data Fig. 8e). The substrate orientation and binding site architecture of Anc^Int^ in the absence of alkali cations were nearly identical to Na^+^/aspartate-bound Glt_Ph_ (Fig. 4d,k). The NMD motif is flipped in with the M311_289_ side chain pointing into the substrate-binding pocket and forming van der Waals interactions with l-aspartate, which is coordinated by side chains of conserved T314_292_, D394_366_, R397_369_ and N401_373_ (Fig. 4k). R397_369_ is the principal residue interacting with the side-chain carboxylate of l-aspartate and D394_366_ coordinates the amino group; these residues determine the specificity of the transporter for the acidic over neutral amino acids and dicarboxylates, respectively^58–60^. N401_373_, one of the most conserved residues throughout the evolution of glutamate transporters, coordinates the main chain carboxylate of l-aspartate. At the current resolution, the architectures of all three Na^+^-binding sites in Anc^Int^ are identical to Na^+^/aspartate-bound Glt_Ph_ (Fig. 4n), although Na^+^ is absent.

Our results suggest that ligand-free Anc^Int^ spontaneously populates the high-affinity state with the correctly formed substrate-binding and ion-binding sites, facilitating substrate binding without Na^+^ binding. In contrast, this is a high-energy state in Glt_Ph_ and other Na^+^-coupled transporters, only accessible upon Na^+^ binding.

### Allosteric mutations turn ion coupling back on

**Fig. 5 |:**
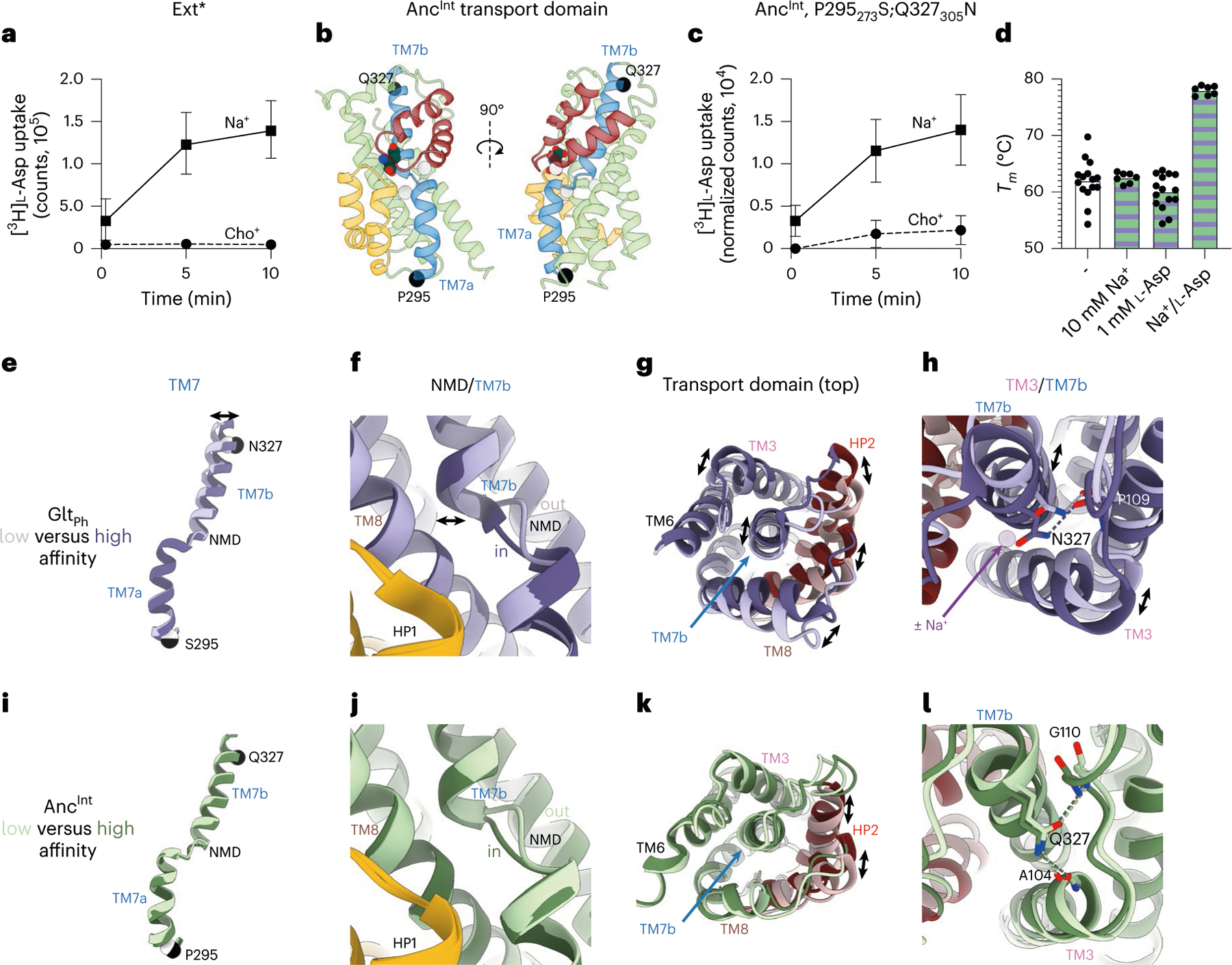
TM7 is the allosteric determinant of Na^+^ coupling. **a**, [^3^H]l-aspartate uptake of Ext* in the presence (filled squares) or absence (filled circles) of a Na^+^ gradient, with the same reaction setup as in Fig. 2c. Error bars are the means ± s.d. of three independent experiments, each the average of at least two technical replicates; error bars not shown are smaller than the displayed symbol. **b**, Transport domain of l-aspartate-bound Anc^Int^, with unoccupied Na^+^-binding sites shown as light-gray spheres, TM7 in blue and P295_273_ and Q327_305_ as black spheres. **c**, [^3^H]l-Aspartate uptake of Anc^Int^ P295_273_S and Q327_305_N with the same reaction setup as Figs. 2c and 4a. Error bars are the means ± s.d. of three independent experiments, each the average of at least two technical replicates. In each individual experiment, the first time point in choline chloride conditions (15 s) was set to zero. **d**, *T*_m_ of Anc^Int^ P295_273_S and Q327_305_N in buffer alone (ligand-free), 10 mM NaCl, 1 mM l-aspartate or both NaCl and l-aspartate. Replicates are from two independent experiments, each with several technical replicates. **e**, Comparison between the low-affinity and high-affinity Glt_Ph_ (**e**–**h**) or Anc^Int^ (**i**–**l**) structures aligned on the rigid HP1 (Glt_Ph_ residues 258–292). Darker structures are the high-affinity states (Na^+^/aspartate-bound Glt_Ph_ (PDB 7RCP) and aspartate-bound Anc^Int^), with the NMD loop positioned toward the substrate-binding site. Lighter structures are ligand-free Glt_Ph_ and low-affinity ligand-free Anc^Int^, with the NMD loop positioned away from the substrate-binding site. All residue numbering corresponds to Glt_Ph_ numbering. **e**,**i**, TM7 alone with P295_273_ and Q327_305_ shown as white and black spheres in low-affinity (**e**) and high-affinity (**i**) states; view corresponds to the rotated (right) view in Fig. 4b. **f**,**j**, A close-up view of the NMD loop (**f**) and the start of TM7b (**j**). Note the larger shift of TM7b in Glt_Ph_ than Anc^Int^. **g**,**k**, a view of the transport domain from the extracellular space showing larger packing differences between low-affinity (**g**) and high-affinity (**k**) conformations in Glt_Ph_. **h**,**l**, Close-up views of [N/Q]327_305_ in TM7 interacting with the top of TM3 in Anc^Int^.

The amino acid sequence changes underlying the transition from Na^+^ coupling in Anc^Na^ to independence in Anc^Int^ are allosteric and lie outside the ion-binding sites. Anc^Na^ and Anc^Int^ are 78% identical over the transport domain sequence (Extended Data Fig. 4c), with a difference in 26 amino acids and PPs above 0.9 in at least one of the ML sequences (Extended Data Fig. 4d). The changes, however, are subtle, making it difficult to pinpoint those responsible for gaining independence from Na^+^ ions. Our phylogenetic tree shows that the evolution from Na^+^-coupled to uncoupled function occurred through several ancestral intermediates between Anc^Na^ and Anc^Int^, which might have gradually acquired the mutations. To narrow down the search for changes necessary for the functional switch, we examined the ancestor immediately preceding Anc^Int^, Anc*, and a present-day transporter most homologous to Anc*, Ext* (KJS87745.1) (Fig. 1d). Anc* and Ext* showed robust Na^+^-coupled binding and uptake, respectively, suggesting they are Na^+^ coupled (Fig. 5a and Extended Data Fig. 9).

There are differences in 18 amino acids between Anc* and Anc^Int^ (95.6% identical), and only two of these are located in the transport domain and have high PPs: S295_273_P (0.969) and N327_305_Q (0.996). These residues are positioned at the beginning and end of TM7, respectively (Fig. 5b). We hypothesized that the reverse substitutions in Anc^Int^, P295_273_S and Q327_305_N, would be sufficient to switch ion coupling back on. Indeed, in proteoliposomes, the mutant exhibited Na^+^-dependent concentrative l-aspartate transport (Fig. 5c). In the thermal shift assays, l-aspartate stabilized the mutant when added with Na^+^ ions but not without them (Fig. 5d and Supplementary Fig. 7). Na^+^ alone induced a deeper and sharper first-derivative trough, consistent with ions binding and increasing the cooperativity of the unfolding process. Both substitutions appear to be necessary, as single mutants showed changes in the melting curves suggesting that the substrate binds without Na^+^ (Extended Data Fig. 9).

We also collected a cryo-EM dataset on Anc^Int^ P295_273_S;Q327_305_N in ligand-free conditions. At the achieved resolution of 5.0 Å, we could not model side chains and the NMD loop; nevertheless, the density of all observed structural classes aligned well with the TM3, TM7b, HP2 and TM8a regions of the low-affinity but not the high-affinity structures of Anc^Int^ (Supplementary Fig. 8). Together, these results suggest that substitutions can rescue Na^+^ coupling in Anc^Int^ by shifting the equilibrium of the ligand-free transporter back to the low-affinity state, thereby restoring coupled substrate and Na^+^ binding. Interestingly, introducing the forward substitutions (S295_273_P and N327_305_Q) into Anc^Na^ did not turn off Na^+^ coupling (Extended Data Fig. 9). Furthermore, introducing these substitutions in Anc* and Ext* resulted in a misfolded, unstable protein. Thus, TM7 mutations are necessary but insufficient to turn ion coupling off. Additional mutations might be required to compensate for negative fitness effects and shift energetics to complete uncoupling.

We compared the low-affinity and high-affinity states of Na^+^-coupled Glt_Ph_ (Fig. 5e–h) and uncoupled Anc^Int^ (Fig. 5i–l) to understand how allosteric mutations might affect their equilibrium. TM7 runs through the core of the transport domain with the NMD motif at its center (Fig. 5b,e,i). In Na^+^-coupled transporters Glt_Ph_ and human EAAT3, TM7b shows a displacement in the low-affinity ligand-free state compared to the high-affinity Na^+^-bound state (Fig. 5e,f and Supplementary Table 1), translating into repacking of the surrounding TM3, TM6, TM8 and HP2 (Fig. 5g,h). Packing differences in this region affect Glt_Ph_ affinity for l-aspartate and are, therefore, allosterically coupled to the changes at the binding site^3,61,62^. In contrast, TM7b of Anc^Int^ shows minimal tilt changes and less helical repacking in the low-affinity state compared to the high-affinity state (Fig. 5i–k), suggesting that S295_273_P and N327_305_Q substitutions stabilize the high-affinity state. P295_273_ is in the loop preceding TM7a and might rigidify the loop compared to serine (Fig. 5b,i). Q327_305_ is the last residue of a highly conserved F-[I/V]-A-[N/Q] signature sequence motif in TM7b^63^ (Fig. 5b,i). Q327_305_ hydrogen-bonds to the top of TM3 (Fig. 5l), perhaps stabilizing TM7–TM3 interactions. By contrast, N327_305_ in Glt_Ph_ is too short to reach TM3 (Fig. 5h). These analyses suggest that the structural flexibility of the central TM7 determines the equilibrium between the transporter states, and a more rigid TM7 in Anc^Int^ tightly packed to surrounding helices might eliminate the need for Na^+^ binding to achieve the high-affinity state.

## Discussion

Na^+^-coupled glutamate transport is one of the most thoroughly characterized ion-coupled transport mechanisms, where Na^+^ binding is inextricably linked to substrate binding. Yet, how these binding sites are allosterically coupled, and thus the design principles of Na^+^-coupled transporters, were unknown. We gained unprecedented insights into this fundamental question by examining the evolution of prokaryotic glutamate transporters using phylogenetics and APR. Surprisingly, the transition from powering transport with Na^+^-to-H^+^ gradients occurred through an intermediate ancestral transporter Anc^Int^, in which l-aspartate binding and transport were uncoupled from Na^+^ binding. Our reconstructed ancestors seem to show slow transport compared to extant transporters, although we did not systematically study this. Reconstructed ancestral proteins are generally more thermostable than extant proteins, which is an established artifact of APR^4,64^. This thermostability might manifest as slow transitional kinetics and slow transport rates.

**Fig. 6 |:**
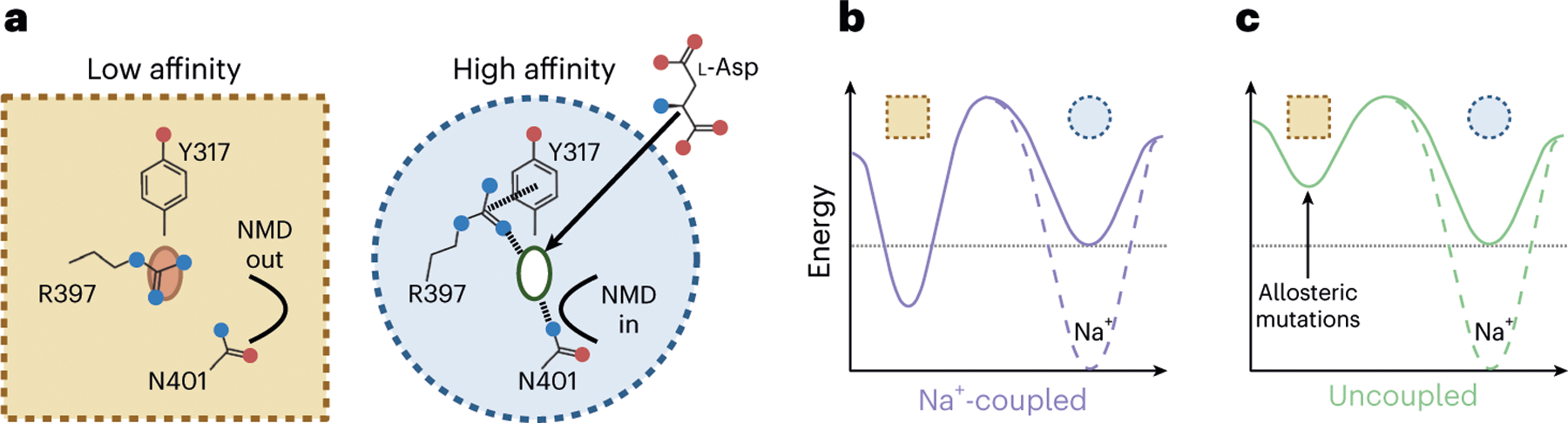
Evolution of the transporter energy landscape controls Na^+^ coupling. **a**, The low-affinity (yellow square) and high-affinity (blue circle) binding configurations highlight changes in R397, N401 and the NMD loop conformations within the binding site. The square and circle represent the differences in the transport domains outside the binding sites allosterically coupled to the conformations of R397, N401 and the NMD loop. The dotted ovals represent local configurations that are unsuitable (red) or ready (green) to coordinate l-aspartate (green solid oval). **b**,**c**, The hypothetical transporter energy landscapes modulated by Na^+^ binding of Na^+^-coupled (**b**) and uncoupled (**c**) transporters. Solid lines represent the relative energies of the low-affinity and high-affinity states in the absence of Na^+^; the dashed line represents the changes upon Na^+^ binding. Relative to the high-affinity state (blue circle), the energy of the low-affinity state (yellow square) in Na^+^-coupled transporters is lower than in uncoupled transporters because of allosteric mutations outside the binding sites. In Na^+^-coupled transporters, Na^+^ binding lowers the energy of the high-affinity state, promoting substrate binding. In Na^+^-uncoupled transporters, Na^+^ binding is unnecessary. This image was created with BioRender.com.

Unlike Na^+^-coupled homologs, Anc^Int^ does not require Na^+^ to form high-affinity substrate-binding and ion-binding sites. Thus, we propose that the relative free energies of the low-affinity and high-affinity states are the linchpin of ion coupling (Fig. 6a–c). In Na^+^-coupled transporters, the free energy of the low-affinity conformation is much lower than the high-affinity conformation (Fig. 6b). The substrate-binding energy alone is insufficient to overcome the penalty, and Na^+^ binding pays the energetic price, stabilizing the state. In uncoupled Anc^Int^, the low-affinity state has a similar or higher energy, and the high-affinity state is populated without ions (Fig. 6c). Na^+^ ions can still bind, possibly with higher affinity because of preformed Na^+^ sites. However, they are unnecessary for substrate binding.

We found two high-probability allosteric mutations between uncoupled Anc^Int^ and the previous ancestor inferred to be Na^+^ coupled. In Anc^Int^, these were sufficient to restore Na^+^-coupled binding and transport, likely by stabilizing the low-affinity state. However, it seems likely that before this switch, transporters accumulated other neutral epistatic mutations during the evolution from Na^+^-coupled Anc^Na^ to Anc^Int^, which gradually increased the relative energy of the low-affinity state. Then, the final two changes were sufficient to make the high-affinity state accessible and function independent of Na^+^ (Fig. 6c). Such allosteric alterations of energy landscapes might underlie the evolutionary diversification of many proteins^65–68^. This mechanism explains how a few mutations can turn off Na^+^ coupling without requiring several mutations to eliminate the three Na^+^-binding sites, which would diminish the transporter’s ability to bind substrate and overall cellular fitness.

Was the transition to uncoupled substrate binding a random evolutionary event or an adaptation required for organismal fitness? While functional studies of Anc^Int^ descendants will need to be carried out, most are unlikely to be ion coupled according to the lack of Na^+^-coupled or H^+^-coupled signature residues. Transporter copies or transporters from different families with similar functions could compensate for the loss of ion-coupled transport^69^, which still raises the question of the physiological relevance of uncoupled transporters. Examining the gene neighbors of uncoupled transporters revealed that many are adjacent to enzymes involved in amino acid metabolism; we observed no similar preferences in the Na^+^-coupled or H^+^-coupled gene neighbors (Extended Data Figure 10). Thus, uncoupled transporters might mediate amino acid uptake paired with their enzymatic use, ultimately maintaining the inward concentration gradient required for continuous uncoupled transport. Such pairing would be reminiscent of glucose transporters, which pair uncoupled transport with subsequent glucose metabolism, akeyinterplayforinsulinsecretion^70^.

In summary, we show that phylogenetic analysis and APR can uncover the evolutionary trajectory of functional diversification in transporters and pinpoint amino acid changes that alter their function. Comparative studies of ion-coupled and ion-independent proteins show that ion coupling can be allosterically regulated and turned on and off by mutations and perhaps environmental changes, which reshape the energy landscape of the transporter. We suggest that drugs could also allosterically modulate the extent of ion and substrate coupling in secondary active transporters, allowing a novel inhibitory modality in which transport is not abolished but its concentrative capacity is diminished. Such drugs could offer advantages in cases where complete inhibition might be harmful.

## Methods

### Sequence retrieval, curation and alignment

We designed a workflow to construct ML phylogenies capturing the Na^+^-coupled to H^+^-coupled (Ext^Na^ to Ext^H^) evolutionary transition on the basis of previous APR studies^47^ (Extended Data Fig. 2). Biochemically characterized Ext^H^ proteins feature signature sequence substitutions in the Na^+^-binding sites compared to Ext^Na^ transporters^2,25,50^: [T/S]92[A/L/I/V/M/F], D405N and M311L (numbering is based on Ext^Na^ archaeal transporter Glt_Ph_) (Fig. 1c). Putative Ext^H^ genes carrying these signature residues are widespread throughout bacterial phyla. We constructed a maximum-parsimony tree of all bacterial glutamate transporter homologs and found that putative Ext^H^ transporters cluster together regardless of phylum (Extended Data Fig. 3a). Although we cannot exclude the existence of H^+^-coupled transporters with completely different signature residues, we infer that the emergence of Ext^H^ was a single-gene duplication event, after which Ext^H^ genes spread through horizontal gene transfer.

Aiming to have a sequence set with a size amenable to APR, we retrieved 20,000 nonredundant Bacillota/Firmicutes sequences using PSI-BLAST (National Center for Biotechnology Information) because Ext^H^ sequences were predominantly found in this phylum. We used WP_011876993.1 as the original query, which we estimated would be close to both Na^+^-coupled and H^+^-coupled transporters^71,72^. After clustering by 99% using CD-HIT^73^, removal of sequences with ambiguous amino acid assignment and alignment by MUSCLE v5 (ref. 74), a maximum-parsimony tree was generated by MP-boot^75^ with the remaining 9,891 sequences. Subsets of sequences were selected on the basis of proximity to sequences with signatures of H^+^ coupling and nearby sequences up until those with signature Ext^Na^ residues (Extended Data Fig. 3b), resulting in 5,249 sequences. The remaining sequences were clustered to 90% using CD-HIT and the resulting 2,539 sequences were manually curated to exclude sequences with major gaps or insertions. Archaeal Glt_Ph_ (UniProt O59010) was included to be the tree root. The final sequence set (1,410 sequences) was aligned by MAFFT-DASH, which incorporates information from Protein Data Bank (PDB) structures into the alignment matrix^76^. Finally, variable loops and tails were removed and the resulting alignment was manually adjusted to account for poorly aligned regions.

### Phylogenetic analysis, ancestral protein reconstruction and construct design

ML trees and ancestral sequences were generated using IQ-TREE 2.1.4-beta (ref. 77). Evaluation of all potential evolution models and rate heterogeneity parameters using ModelFinder revealed that LG + F + R was the best protein evolution model^78–80^. FreeRate (F) heterogeneity considers site-specific differences in evolutionary rate, relaxing the constraint of equal site probabilities imposed by discrete gamma rate heterogeneity. The number of possible rate categories that could be assigned to a site (R) was empirically determined for each run using ModelFinder to query a range of 10–20.

As with other APR studies of microbial proteins, our tree topology is not reconciled to the microbial species tree, as this has poor confidence especially at lower taxonomic ranks^81^. We chose the outgroup-based approach of rooting the tree, using archaeal Glt_Ph_ as the outgroup because of the evolutionary distance between bacteria and archaea; Glt_Ph_ was also distant enough to the ingroups and did not introduce any long-branch attraction artifacts. Most archaeal transporters contain all Na^+^-coupling signature residues (Extended Data Fig. 3c), suggesting that the origin of glutamate transport was sodium coupled and the divergence into sodium-independent exchangers and subsequent proton-coupled transporters occurred after the divergence of bacteria and archaea. After performing over 60 replicate tree runs, we found that the best Firmicutes tree yielded robust UFBoot2 values, ranging between 91% and 100% for the Ext^H^ node (Fig. 1d). When we repeated the analysis for the Proteobacteria phylum, the best tree had 5–87% UFBoot2 values for the equivalent nodes, reflecting nonreproducible trees. Thus, we used the Firmicutes tree for all subsequent analyses.

The run yielding the best tree, as determined by log likelihood, was inspected for long branches, defined as >0.7 substitutions per site. These were removed and, in the second round of tree-searching with the final dataset of 1,153 sequences, the analysis was repeated with 254 independent tree search runs. The final tree had the best-fit model of LG + F + R14, chosen according to the Bayesian information criterion. Bootstrap values were determined by UFBoot2 using 1,000 replicates^45^. Ancestral sequences were reconstructed from the best tree using the IQ-TREE implementation^77^ based on Yang’s marginal reconstruction method^82–84^. The ML sequences were generated using custom Python scripts, either written manually or generated using ChatGPT. The final ML amino acid sequence for each node was constructed using the amino acid with the highest PP at every site. All tree figures were generated using TreeViewer^85^. Visual representations of sequence alignments and PPs were made using WebLogos^86^.

### Protein expression and purification

Because poorly aligned loops and tails are not reconstructed in our APR analysis, we had to graft them in for expression constructs. We took comparable loops and tails from an extant sequence close to Anc^Int^ (KJS87745.1); the 3L4 has two lysines that were substituted to histidines (K105H;K109H) to prevent potential proteolysis. All protein constructs were codon-optimized for *E. coli*, synthesized (GenScript) and cloned into a modified pBAD vector containing a C-terminal thrombin cleavage site followed by either an ALFA peptide tag^87^ or a C-terminal thermostable GFP specialized for membrane proteins^49^. An additional Twin-Strep tag and a 10×His tag were added to the C terminus of these constructs, all separated by glycine–serine (GSSS) linkers. The Glt_Ph_ construct used was the ‘CAT7’ construct, with seven histidines previously introduced for stability^13^. Vectors were transformed into *E. coli* BL21(DE3) or DH10B cells (New England Biolabs, C2527; Invitrogen, 12331013), cells were grown in 2×YT medium and expression was induced by adding 0.1% arabinose at 0.8–1.0 OD_600_. Proteins were expressed for 2–3 h at 37 °C. Membranes were purified as previously described for Glt_Ph_, with all steps being performed at 4 °C (ref. 3). Briefly, cells were broken using a high-pressure cell disruptor (Avestin) for three passes with cooled water continuously applied. The resulting lysate was spun at 17,400*g* for 15 min and the supernatant was spun at 186,000*g* for 1 h. The resulting membrane pellet was homogenized in 20 mM HEPES–NaOH pH 7.4, 10 mM EDTA and 10% sucrose. Finally, the slow and fast spins were repeated and the resulting pellet was frozen at −80 °C.

All protein purification steps were performed at 4–8 °C. Crude membranes were homogenized in 20 mM HEPES–NaOH pH 7.4, 200 mM NaCl and 1 mM monopotassium l-aspartate; solubilization was initiated by the addition of 40 mM *n*-dodecyl-β-d-maltopyranoside (DDM; Anatrace) and the solubilizing membranes were gently rocked for 2 h. Over the course of our experiments, we noticed that tags were being nonspecifically proteolyzed upon solubilization; to reduce this, we added 1 mM PMSF and 1 mM EDTA before adding DDM. After solubilization, samples were clarified by high-speed ultracentrifugation at 186,000*g* for 1 h.

For ancestral constructs, the supernatant was applied to a pre-packed 1-ml Streptactin XT 4Flow high-capacity column (IBA Biosciences) at a constant flow rate of 0.5–1 ml min^−1^. The resin was washed until the ultraviolet absorbance at 280 nm (UV_280_) was flat, corresponding to 14 column volumes of 20 mM HEPES–NaOH pH 7.4, 200 mM NaCl, 1 mM l-aspartate and 1 mM DDM. Subsequently, the resin was eluted with the same buffer containing 50 mM biotin (IBA Biosciences) and proteins were concentrated.

For Glt_Ph_, the supernatant was applied to a 1-ml HisTrap HP (Cytiva) at a constant flow rate of 1 ml min^−1^. The resin was washed until the UV_280_ was flat, corresponding to 30 column volumes of 20 mM HEPES–NaOH pH 7.4, 200 mM NaCl, 1 mM l-aspartate, 40 mM imidazole and 1 mM DDM. Subsequently, the resin was eluted with the same buffer containing increased imidazole (250 mM) and proteins were concentrated.

### Reconstitution into proteoliposomes

Unilamellar liposomes were prepared essentially as previously described^3^. Briefly, a 3:1 (w/w) ratio of *E. coli* polar lipids and egg phosphatidylcholine (Avanti Polar Lipids) was dried, hydrated in 50 mM HEPES–Tris pH 7.4 and 200 mM KCl at a final concentration of 5 mg ml^−1^, flash-frozen and stored at −80 °C. On the day of the reconstitution, liposomes were thawed, extruded through a 400-nm filter 13 times and destabilized with Triton X-100 using a 1:2 (w/w) ratio of Triton X-100 to lipid. For the counterflow and Na^+^-coupled uptake experiments, GFP-free proteins were purified by size-exclusion chromatography (SEC) using a Superdex 200 Increase 10/300 column (Cytiva) in 20 mM HEPES–NaOH pH 7.4, 200 mM NaCl, 1 mM l-aspartate and 1 mM DDM; proteins were subsequently concentrated using a 100-kDa-cutoff concentrator. Proteins in all comparative experiments (Fig. 2d–g) were purified and reconstituted alongside one another. To form proteoliposomes, the purified protein was mixed with destabilized liposomes (1:100, w/w) for 30 min; then, detergent was removed with BioBeads SM-2 (Bio-Rad) at 80 mg ml^−1^, which were applied twice at 22 °C for 2 h, once overnight at 4 °C and once more at 22 °C for 2 h.

### Proteoliposome uptake experiments

After detergent removal with BioBeads, proteoliposomes underwent the same general procedure for buffer exchange and concentration, regardless of experiment. Proteoliposomes were exchanged into the appropriate buffer using a PD-10 column (Cytiva), flash-frozen in liquid N_2_, thawed, concentrated by ultracentrifugation (100,000*g* for 1 h), resuspended to a final concentration of 50 mg ml^−1^, flash-frozen and stored at −80 °C. On the day of the experiment, the proteoliposomes were thawed and extruded through a 400-nm filter 23 times. To minimize sample dilution in the extruder, the proteoliposome volume was no less than 100 μl. All subsequent experiments were performed at 30 °C.

For uptake experiments, liposomes were prepared as described above to a final concentration of 50 mg ml^−1^ in 20 mM HEPES–Tris pH 7.4, 200 mM KCl and 100 mM choline chloride. To initiate Na^+^-driven uptake in the presence of a negative membrane potential (−102 mV), liposomes were diluted 1:100 in 20 mM HEPES–Tris pH 7.4, 2 mM KCl, 198 mM choline chloride, 100 mM NaCl, 0.9 μM valinomycin and 1 μM l-aspartate. The l-aspartate was a mixture of cold monopotassium l-aspartate (Research Products International) and [^3^H]l-aspartate (PerkinElmer or American Radiolabeled Chemicals). For negative controls without the Na^+^ gradient, NaCl was replaced with choline chloride.

To stop the reactions, 200 μl of reaction mixture (100 μg of liposomes) was diluted in 2 ml of ice-cold quenching buffer (20 mM HEPES–Tris pH 7.4 and 200 mM lithium chloride). The quenched reaction was immediately filtered through a 0.22-μm filter membrane (Millipore Sigma) and washed three times with 2 ml of quenching buffer. Washed membranes were inserted into scintillation vials and the membranes were soaked overnight in 5 ml of Econo-Safe counting cocktail. Radioactivity in liposomes was measured using an LS6500 scintillation counter (Beckman Coulter). Technical replicates from an individual reconstitution were taken in duplicate and averaged and at least two separate reconstitutions were taken per point. Data were analyzed using GraphPad Prism 10.

### Proteoliposome counterflow experiments (sodium-dependent)

For counterflow experiments, the proteoliposomes after BioBead detergent removal were similarly exchanged in 20 mM HEPES–Tris pH 7.4, 200 mM KCl and 100 mM choline chloride using PD-10 columns. The exchanged liposomes were then separated into tubes for sodium conditions and choline conditions. For sodium conditions, an equivalent buffer containing 100 mM sodium chloride instead of choline chloride was added after exchange so that the final sodium concentration was 10 mM and the final choline concentration was 90 mM. Choline conditions were treated similarly, with the original choline buffer instead of the sodium buffer. A final concentration of 0.5 mM monopotassium l-aspartate was added to both conditions. Finally, the liposomes were flash-frozen and thawed twice, concentrated by ultracentrifugation (100,000*g* for 1 h), resuspended in the same internal buffer at 50 mg ml^−1^, flash-frozen and stored at −80 °C as described in the uptake experiments.

On the day of the experiment, proteoliposomes were thawed and freshly extruded, as described in the uptake experiments. Counterflow was initiated by diluting the liposomes 1:100 in an external buffer equivalent to the internal buffer, except that the cold 0.5 mM l-aspartate was replaced by 0.5 μM [^3^H]l-aspartate (American Radiolabeled Chemicals). Negative controls had an additional 0.5 mM cold l-aspartate in the external buffer. Reactions proceeded for the indicated time and were stopped and measured as described in the uptake experiments. Data were analyzed using GraphPad Prism 10.

### Proteoliposome counterflow experiments (pH-dependent)

Proteoliposomes after BioBead detergent removal were exchanged in 50 mM potassium phosphate (KH_2_PO_4_–K_2_HPO_4_) buffer plus 0.5 mM l-aspartate (pH 6, 7 or 8) by three rounds of resuspension, two freeze–thaw cycles and ultracentrifugation (100,000*g* for 1 h). These were performed so that the total internal buffer exchange was greater than 99.5%. After the final round, proteoliposomes were resuspended to a final concentration of 25 mg ml^−1^, flash-frozen and stored at −80 °C. On the day of the experiment, proteoliposomes were thawed and freshly extruded as described in the uptake experiments. Counterflow was performed as in the sodium-dependent counterflow experiments, except the dilution factor was 1:50 and the quenching buffer was 50 mM MES–KOH buffer pH 5.5. Data were analyzed using GraphPad Prism 10.

### nanoDSF

All steps were performed on the same day of the protein solubilization and purification because we observed variance in results depending on the freshness of the protein. We opted to use GFP-tagged ancestral proteins because the *T*_m_ of the fused GFP is greater than 90 °C (ref. 49), which does not interfere with the *T*_m_ values observed in our experiments. After affinity purification, the proteins with C-terminal GFP were concentrated using a 100-kDa-cutoff concentrator (MilliporeSigma). To remove ligands, the protein was dialyzed using a PD MiniTrap G-25 (Cytiva). For Anc^Na^ and Anc^Int^ P295_273_S;Q327_305_N, this buffer was 20 mM HEPES, 200 mM NMDG-Cl pH 7.4 and 1 mM DDM; for Anc^Int^, this buffer was 50 mM KH_2_PO_4_–K_2_HPO_4_ pH 7 and 1 mM DDM. Different buffers were used for optimal first-derivative curves in ligand-free conditions and we observed identical *T*_m_ values in substrate-bound conditions regardless of dialysis buffer composition. The dialyzed, ligand-free protein was concentrated and diluted into the same respective buffers at a final concentration of ~1 mg ml^−1^; for Anc^Int^, the buffer contained 10 mM decyl-maltoside (DM) instead of 1 mM DDM. This was necessary to observe melting of Anc^Int^, presumably by the shorter chain detergent destabilizing the overall protein. Thermal stability in the presence and absence of various potential ligands was measured using a Tycho NT.6 instrument (Nanotemper) and individual *T*_m_ values were calculated by determining the appropriate local minimum in the first derivative of the 350-nm:330-nm ratio. Data were analyzed using GraphPad Prism 10.

### MST

After affinity purification, the proteins with C-terminal GFP were concentrated using a 100-kDa-cutoff concentrator and then SEC-purified on a Superose 6 Increase 10/300 column (Cytiva) in 50 mM KH_2_PO_4_–K_2_HPO_4_ buffer pH 7 and 1 mM DDM. For the MST experiment, monopotassium l-aspartate was serially diluted twofold in water for a total of 16 concentrations. Each aspartate dilution was mixed with in a 1:1 (v/v) ratio of protein samples so that the final protein concentration was 100–300 nM, the appropriate salt was 100 mM, the DDM was 0.4 mM and the KH_2_PO_4_–K_2_HPO_4_ was 10–20 mM. Measurements were taken using Monolith NT.115 Blue/Red MST (Nanotemper), high MST power, a blue detector (for GFP) and premium capillaries (Nanotemper).

For data analysis, obvious outliers caused by aggregates (as determined by instrument software, MO.Control 2) were discarded. The ideal response time for each individual experiment was determined by instrument software and this was always between 1.5–5 s. Individual binding titrations were normalized to fraction bound by *K*_*D*_ fits in MO.Affinity Analysis version 2.3. To obtain the final binding affinity value, the experiments were repeated three independent times, the data points were averaged and the *K*_D_ was calculated from the average values. Data were analyzed using GraphPad Prism 10.

### Cryo-EM sample preparation

Membrane scaffold protein (MSP1E3) was expressed and purified as previously described^88^ in the absence of Na^+^ or l-aspartate. A 3:1 (w/w) ratio of *E. coli* polar lipids and egg phosphatidylcholine (Avanti Polar Lipids) was dried, hydrated in 50 mM HEPES–Tris pH 7.4 and 200 mM KCl and solubilized in 72 mM DDM at final lipid concentration of 18 mM as previously described^15,88^.

For Anc^Int^, GFP-free transporter constructs were affinity-purified, concentrated and mixed with MSP1E3 and solubilized lipids (both containing no Na^+^ or l-aspartate) at a molar ratio of 1.5:1:50, aiming for a final concentration of lipid greater than 5 mM. This mixture was incubated at 22 °C for 30 min; then, 350 mg of BioBeads SM-2 (Bio-Rad) per ml of mixture (w/v) was added for 2 h at 22 °C with gentle shaking and another round of BioBeads was applied overnight at 4 °C with gentle shaking. The resulting MSP1E3 and protein preparations were cleared by ultracentrifugation at 100,000*g* for 30 min. The supernatant was applied to a Superose 6 Increase 10/300 column in 20 mM HEPES pH 7.4, 100 mM NMDG-Cl and the peak fractions were concentrated to 7–9 mg ml^−1^, as measured by absorbance at 280 nm (A_280_). For the Anc^Int^ substrate-bound dataset, 1 mM l-aspartate was added to the sample just before freezing. Reconstitution efficiency was verified using SDS–PAGE.

Glt_Ph_ was reconstituted into MSP1E3 as previously described^15^. Affinity-purified protein was further purified by SEC (Superdex 200 Increase, 10/300). Peak fractions were concentrated, mixed with MSP1E3 and solubilized lipids at a molar ratio of 0.75:1:50, aiming for a final concentration of lipid greater than 5 mM. This mixture was incubated and treated with BioBeads SM-2 in the exact same fashion as ancestral protein. The resulting MSP1E3 and protein preparations were exchanged in Na^+^-free buffer by three rounds of tenfold dilution and concentration and cleared by ultracentrifugation at 100,000*g* for 30 min. The supernatant was applied to a Superose 6 Increase 10/300 column in 20 mM HEPES–Tris pH 7.4 and 50 mM choline chloride, with the goal of mimicking previously published conditions. The peak fractions were concentrated to 8 mg ml^−1^, as measured by A_280_. Reconstitution efficiency was verified using SDS–PAGE.

To improve particle distribution, fluorinated Fos-Choline-8 (Anatrace) dissolved in water was added to the sample to a final concentration of 1.5 mM. UltrAuFoil R1.2/1.3 300-mesh gold grids (Quantifoil) were glow-discharged at 25 mA for 80 s. Then, 3 μl of protein was applied to each grid, incubated for 20 s under 100% humidity at 4 °C, blotted for 2.5 s under 0 blot force and plunge-frozen in liquid ethane using a Vitrobot Mark IV (Thermo Fisher Scientific).

### Cryo-EM data acquisition and processing

Imaging was performed on a Titan Krios (FEI) equipped with a K3 camera (Gatan) using Leginon software. Specifics of each data collection and each individual map and model are provided in Table 1. Visual summaries of processing workflows and EM validation are provided in Extended Data Fig. 6 and Supplementary Figs. 4–6 and 8.

#### Initial processing pipeline for Anc^Int^.

For all datasets, raw videos were imported into cryoSPARC 4.0–4.4 and subjected to patch motion correction and patch contrast transfer function (CTF) estimation, where the videos were binned twice^89^. Low-quality videos were removed, as determined by CTF fits, ice thickness, intensity, etc. Particles were manually picked to train Topaz^90^, where, for each dataset, the optimal picking model and estimated number of particles per micrograph were empirically determined using the Topaz cross-validation tool in cryoSPARC. After picking and extraction with four binning rounds, an ab initio 3D model was constructed and high-quality particles were sorted using multiple heterogeneous refinement jobs, where ‘good’ particles sorted into the ab initio model class and ‘junk’ particles sorted into three pure noise ‘decoy’ volume classes (created by performing one iteration of ab initio). When greater than 95% of particles converged into the protein volume, the remaining particles were re-extracted to full box size and subjected to 3D classification without alignment and nonuniform (NU) refinement^91^. To maximize the number of particles obtained, the Topaz model was retrained on the clean particle stack after rounds of two-dimensional classification and heterogeneous refinement was repeated with the new particle stack and the high-resolution volume from NU refinement (replacing the ab initio volume). Remaining particles were re-extracted to full box size and subjected to NU refinement.

#### Anc^Int^ ligand-free dataset processing.

Our ancestral protein datasets were highly heterogeneous and poorly symmetric; throughout the processing, we realized that they were reminiscent of other seemingly mobile membrane protein structures^92,93^. To overcome this, we attempted to enrich particles with symmetric features so that we could classify individual protomers. In the ligand-free dataset, we converted particle coordinates to RELION format using PyEM^94^. Particles were re-extracted in RELION using videos motion-corrected and binned twice using RELION’s implementation of MotionCor2 (ref. 95). These particles were reimported into cryoSPARC and refined using NU refinement (*C*_1_); the resulting particle images were converted to RELION format with PyEM. These particles were then subjected to two rounds of Bayesian polishing^96^, classification with heterogeneous refinement and classification with NU refinement (*C*_1_). To remove ‘disordered’ particles, we used 3D classification without alignment (*C*_1_) in cryoSPARC. We further optimized symmetric particles by performing one round of ab initio with *C*_3_ symmetry imposed. Using the best class, we achieved a resolution of 2.8 Å in *C*_3_, which allowed us to resolve individual protomers using symmetry expansion and focused classification using a mask on the transport domain. Each class underwent local refinement masked on the entire protomer (including transport and scaffold domains) using pose and shift Gaussian priors during alignment (standard deviations of 3° and 2 Å).

#### Anc^Int^ substrate-bound processing.

As with the ligand-free dataset, the substrate-bound dataset was similarly heterogeneous and asymmetric. Using the previous approach, we encountered trouble imposing *C*_3_ symmetry, preventing focused classification of individual protomers. During our processing, we realized that we could more efficiently remove disordered particles using outputs from 3D variability (3DVA), where the first variability component reflects this ‘order–disorder’ transition between rigid, easily aligned particles and presumably mobile, difficult-to-align particles. We then took the first frame of the 3DVA video (disordered, bad volume), the last frame of the video (ordered, good volume) and two decoy noise volumes as inputs for heterogeneous refinement. Though a fundamentally similar approach to 3D classification used in the ligand-free dataset, the new approach allowed us to impose *C*_3_ symmetry with good resolutions (3.4 Å). We then symmetry-expanded the particles, performed 3D classification without alignment on the masked transport domain and locally refined the protomers using pose and shift Gaussian priors during alignment (standard deviations of 3° and 2 Å), as with the ligand-free dataset.

#### Glt_Ph_ ligand-free processing.

Raw videos were imported into cryoSPARC 4.4 and subjected to patch motion correction and patch CTF estimation, where the videos were binned twice^89^. Low-quality videos were removed, as determined by CTF fits, ice thickness, intensity, etc. Particles were manually picked to train Topaz^90^, where the optimal picking model and estimated number of particles per micrograph were empirically determined using the Topaz cross-validation tool in cryoSPARC. After picking and extraction, an ab initio 3D model was constructed. After one round of heterogeneous refinement with ab initio volumes, we performed NU refinement (*C*_3_) on the remaining particles, symmetry-expanded the particles, performed 3D classification without alignment on the masked transport domain and locally refined the protomers using pose and shift Gaussian priors during alignment (standard deviations of 3° and 2 Å).

#### Anc^Int^ P295_273_S;Q327_305_N ligand-free dataset processing.

Because of the substantially lower resolution of the dataset, previously described methods to improve the resolution after decoy classification (3DVA and 3D classification without alignment) did not work. Therefore, we immediately performed 3D classification (*C*_3_) after decoy classification. We then performed symmetry expansion and 3D classification without alignment on the masked transport domain.

### Model building and refinement

For model building, maps were sharpened using DeepEMhancer^97^. We generated a homology model of Anc^Int^ in SWISS-MODEL, using an outward-facing state of Glt_Ph_ (PDB 2NWX) as a template^13,98^. The template for Glt_Ph_ was PDB 6UWF (outward-facing) or PDB 6UWL (intermediate outward-facing). The model was adjusted into the density using ISOLDE^99^ and the final model was iteratively real-space refined and validated in PHENIX using the unsharpened density and default parameters^100^. All structural figures were made with ChimeraX^101^.

### Gene neighborhood analysis

For each clade (Ext^Na^, Ext^Int^ and Ext^H^), a sequence similarity network and corresponding genome neighborhood was created using the Enzyme Function Initiative webserver^102^. Transporters without information regarding protein-coding gene neighbors were excluded. A transporter was considered paired to an enzyme if they were within three neighboring genes away from the transporter. Genes were considered to have amino-acid-modifying or dicarboxylate-modifying activity according to Pfam annotation.

### Reporting summary

Further information on research design is available in the Nature Portfolio Reporting Summary linked to this article.

## Extended Data

**Extended Data Fig. 1 |:**
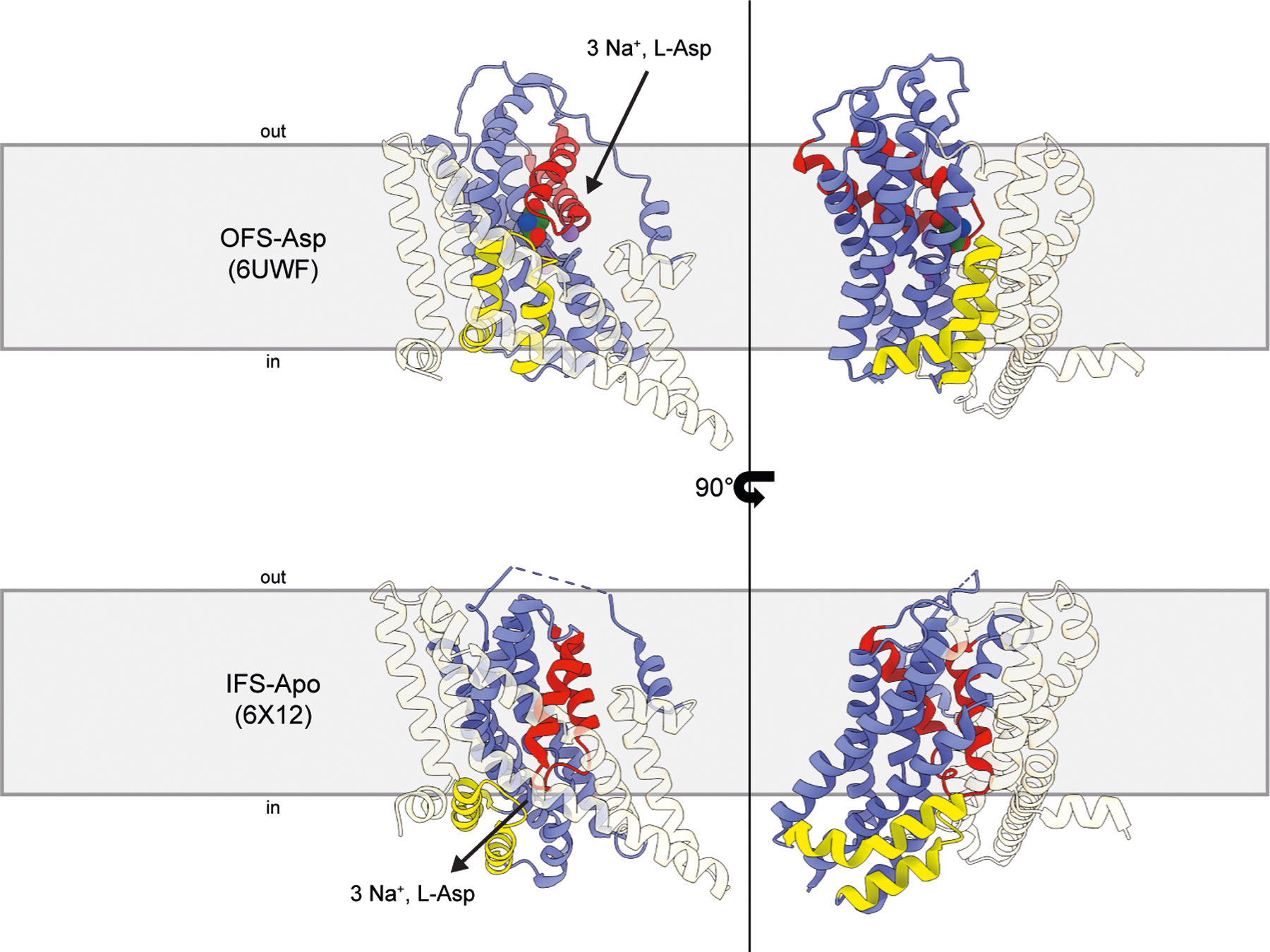
Elevator mechanism of SLC1-type transporters. Glt_Ph_ structures in the substrate-bound outward-facing state (OFS-Asp, PDB 6UWF) and substrate-free inward-facing open state (IFS ligand-free, 6X12). The rigid scaffold domain is in transparent wheat; the mobile transport domain is in blue; HP1 is in yellow; HP2 is in red; purple spheres are Na^+^; green molecule is L-aspartate. In a typical transport cycle, three Na^+^ ions and L-aspartate bind in the outward-facing state, resulting in the closure of the HP2 gate and translocation of the transport domain to the inward-facing state. The HP2 gate then opens, releasing solutes to the intracellular side.

**Extended Data Fig. 2 |:**
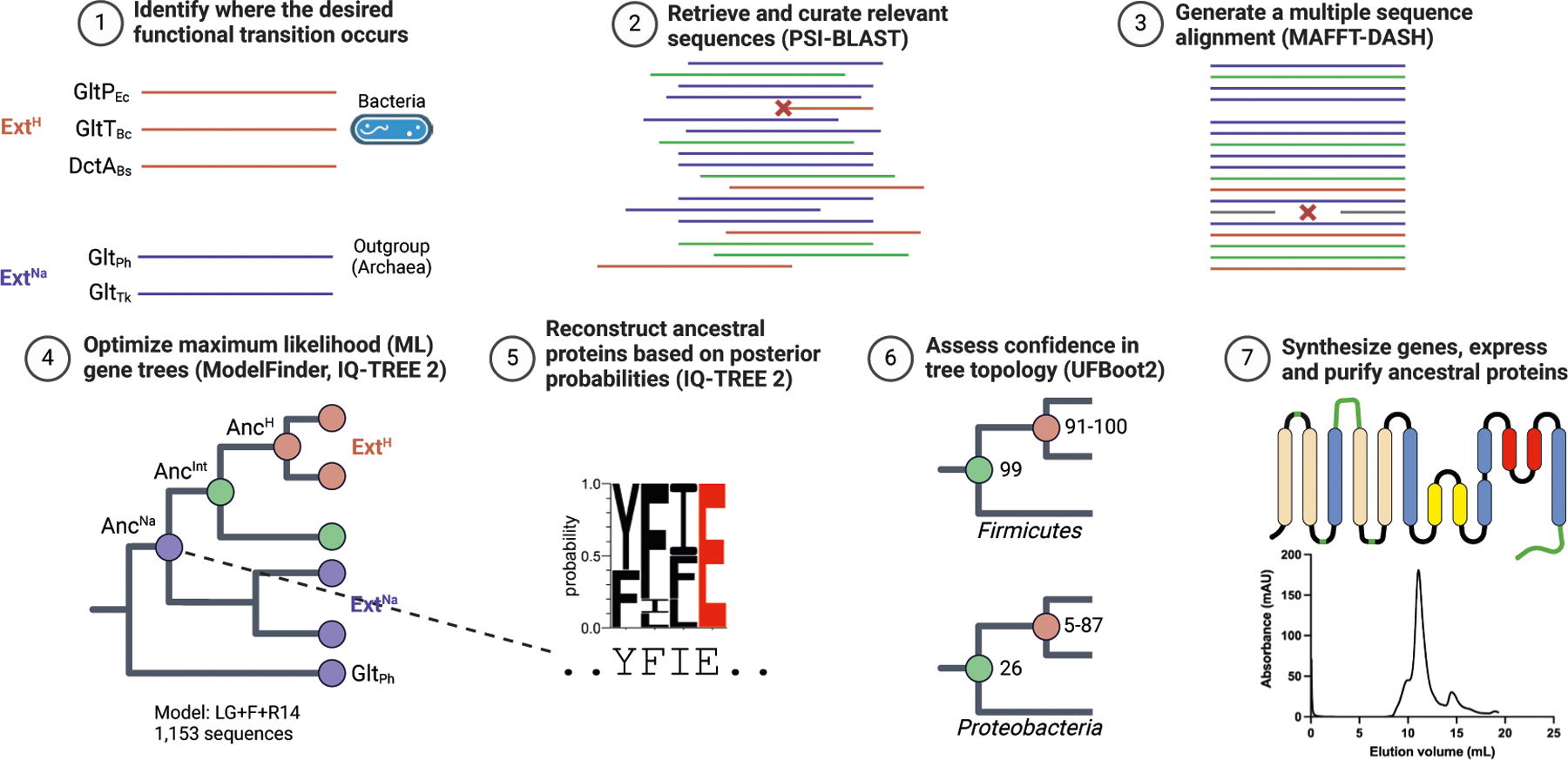
APR workflow to capture Ext^Na^ to Ext^H^ evolutionary transition. The diagram is based on Orlandi et al. (1) Based on previous biochemical and structural characterization of prokaryotic H^+^-coupled (orange; GltP_Ec_, GltT_Bc_, DctA_Bs_) and Na^+^-coupled (purple; Glt_Ph_, Glt_Tk_) transporters, we identified signature residues that helped us sort putative sequences as either Ext^H^ or Ext^Na^. (2) We retrieved homologs of transporters using PSI-BLAST. After clustering and quick sequence alignment, we removed sequences with large gaps or insertions. Finally, we used parsimony tree generation to keep sequences that encompass Ext^H^, Ext^Na^, and intermediate (green) transporters. (3) We carefully aligned the sequences using MAFFT-DASH, which incorporates structural information. Sequences with large gaps or insertions were removed. The remaining sequences were re-aligned, and poorly aligned loops and tails were stripped from the alignment. (4) The final sequence alignment was used as input for ML tree generation, using archaeal Glt_Ph_ as the root. The optimal model of protein evolution was selected using ModelFinder. Sequences that caused long branches were removed, and 254 parallel tree searches were performed to find the optimal branch lengths and topology, resulting in the ML tree with the highest possible likelihood. (5) To reconstruct ancestral sequences, every node was reconstructed using optimized ML parameters and protein evolution model; each site in each node is assigned probabilities for all 20 amino acids, and the highest-probability amino acid at a site is selected for the ML sequence. (6) To determine if a given node is creating a robustly reconstructed ancestor, bootstrap is performed (UFBoot2), which calculates branch supports by resampling the MSA 1000 times and determining the frequency of the branch re-occurring in the trees. Sequence sampling is critical in this step; when we retrieved sequences from *Firmicutes/Bacillota*, we obtained robust UFBoot2 values for Anc^Int^ and descendants, but not when retrieving sequences from *Proteobacteria*. (7) Ancestral sequences are engineered for expression and purification by inserting loops and tails from extant sequences (green), which were not considered during APR due to poor alignment. All relevant references here and elsewhere in the legends are in the Main Text or Methods. Created in BioRender. Boudker, O. (2025) https://BioRender.com/sl3la84.

**Extended Data Fig. 3 |:**
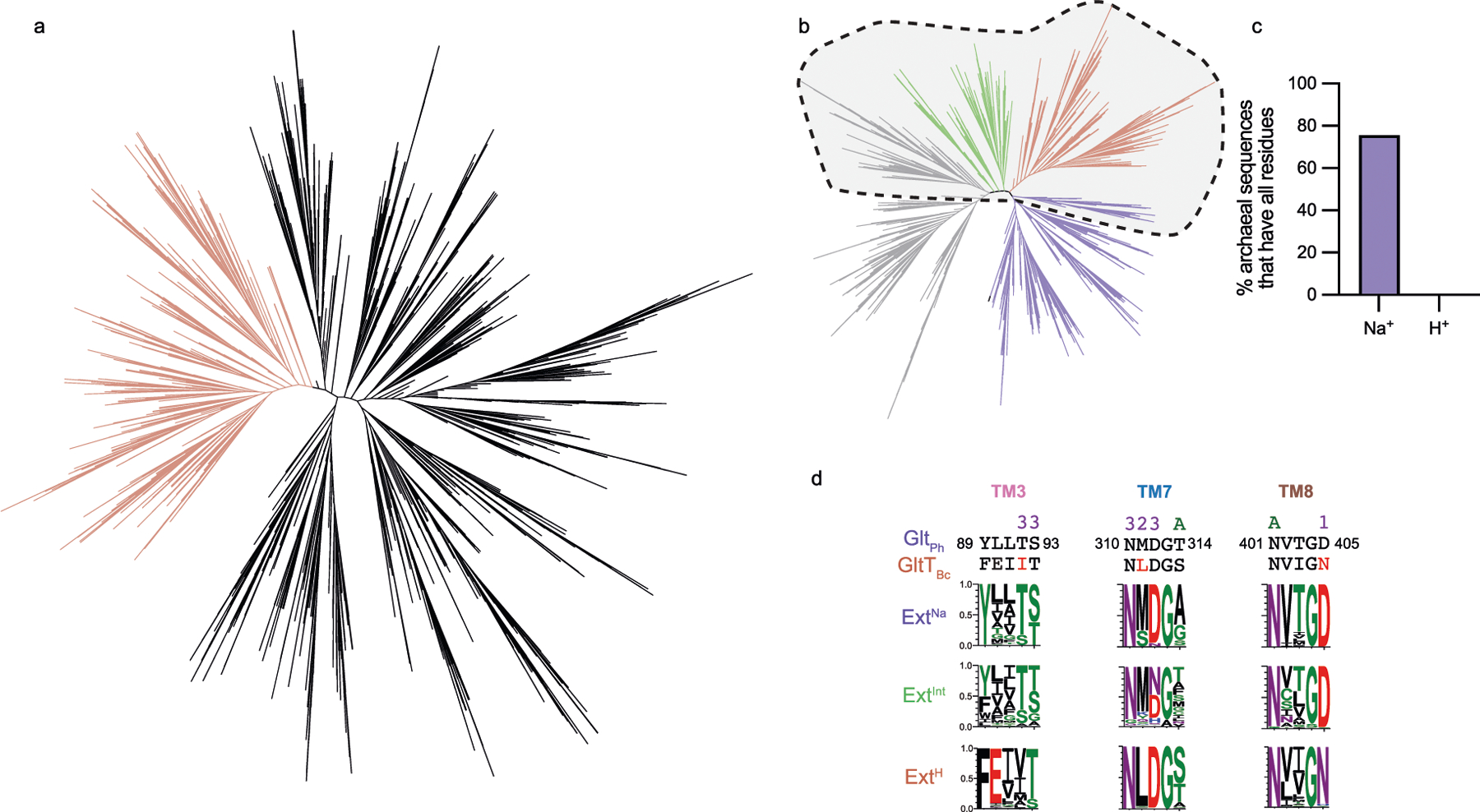
Phylogeny, rooting, and conservation of prokaryotic glutamate transporter homologs. (**a**) Maximum parsimony phylogeny of bacterial glutamate transporter homologs. The clade containing conserved signature residues in H^+^-coupled transporters is colored orange. (**b**) Selection of initial sequences for APR analysis from *Firmicutes/Bacillota* (99% clustered). Purple, green, and orange clades contain representative Ext^Na^, Ext^Int^, and Ext^H^ sequences, respectively. Gray sequences were unassigned in our analysis, and were eventually removed due to potential long-branch attraction artifacts. (**c**) Conservation of Na^+^- and H^+^-coupling signature residues in non-redundant archaeal glutamate transporters. (**d**) Conservation of the Na^+^-binding sites throughout the ML phylogeny. Top: sequences of Na^+^-coupled Glt_Ph_ and H^+^-coupled GltT_Bc_ encompassing Na^+^-binding sites of Ext^Na^. The numbers above the sequences mark side chains in Glt_Ph_ that coordinate Na^+^ sites 1–3; A indicates side chains coordinating L-aspartate. Bottom: WebLogos of the relative amino acid frequencies in Ext^Na^, Ext^Int^, and Ext^H^ clades. The number of sequences per clade is 186, 161, and 786, respectively.

**Extended Data Fig. 4 |:**
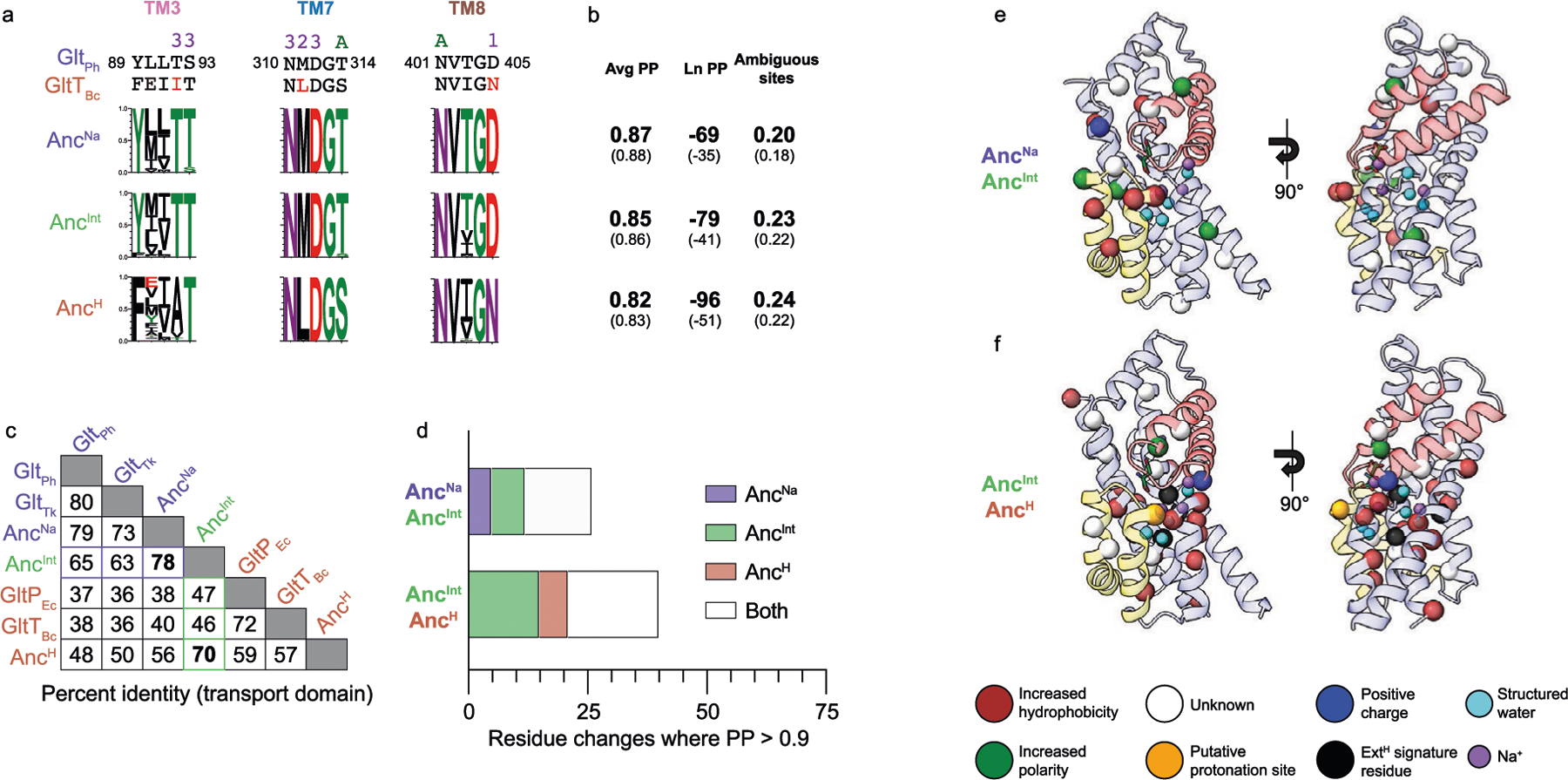
Robustness of the ancestral protein reconstruction. (**a**) Reconstructed ancestral sequences in Na^+^-binding sites of Anc^Na^, Anc^Int^, and Anc^H^. WebLogos visually represent the posterior probability per site in each ancestral sequence. The topmost residue at each site is the ML residue. Glt_Ph_ and GltT_Bc_ sequence alignments are shown above the WebLogos for reference, along with annotations for L-aspartate binding residues (A) or Na^+^-binding residues^1–3^. (**b**) Average posterior probability (PP) per site, the natural logarithm of the total posterior probability of the entire ancestral sequence (Ln PP), and the fraction of the ambiguous sites (defined as two or more amino acids per site with a probability greater than 0.2) in each ancestor. Bold, larger values are for the entire sequence; values in parentheses are for the transport domain alone. (**c**) Pairwise identity (in %) matrix of ancestral and extant transporters. The alignment included only transport domains, defined as Glt_Ph_ residues 78–110, 228–416. (**d**) Visual representation of the changes between two ML ancestral sequences. In each bar, the first segment is the number of changes where the posterior probability is greater than 0.9 in only the first ancestor, that is, a robustly reconstructed site is ambiguously reconstructed in the second ancestor; the second segment is the number of changes where the posterior probability is greater than 0.9 in only the second ancestor, while the site is ambiguous in the first ancestor; the third segment is the number of changes where the posterior probability is greater than 0.9 in both ancestors with both sites confidently reconstructed. (**e, f**) Probable sequence changes between Anc^Na^ and Anc^Int^ (**e**) and Anc^Int^ and Anc^H^ (**f**), mapped onto the transport domain of Glt_Ph_. Bottom: Labeling scheme for residues. Larger spheres represent sequence transitions; smaller spheres represent Na^+^ sites and structured waters observed in Glt_Ph_ (PDB 7RCP). ‘Unknown’ indicates when there is no clear physicochemical difference between the two residues. Ext^H^ signature residues are [T/S]92[A/L/I/V/M], M311L, and D405N; putative protonation site is S279E.

**Extended Data Fig. 5 |:**
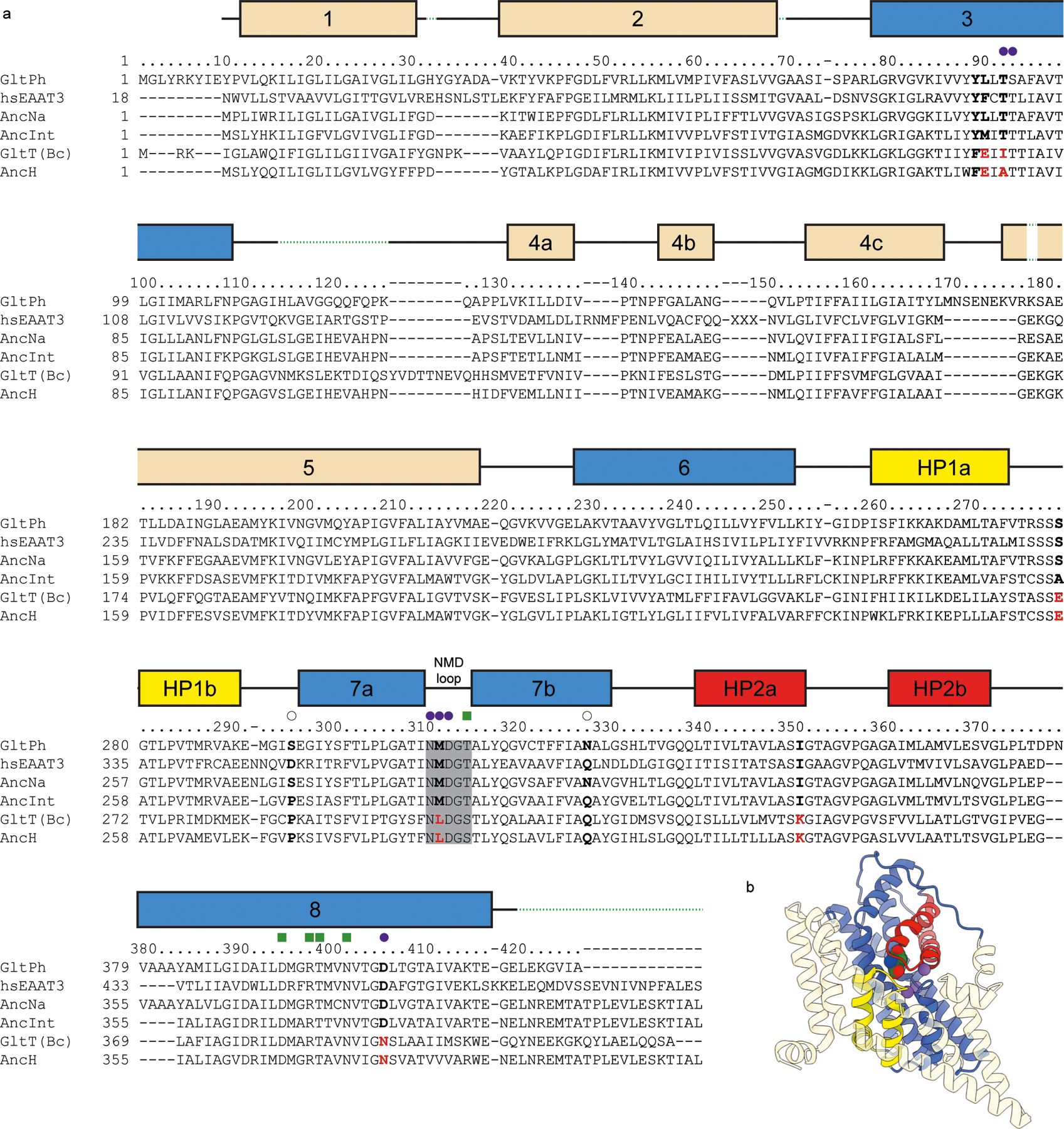
Sequence alignment of extant and ancestral glutamate transporter homologs. (**a**) Alignment sequence numbering corresponds to Glt_Ph_ numbering. Extant sequences are Glt_Ph_ (UniProt O59010), GltT_Bc_ (P24944), and human EAAT3 (P43005). TMs, shown above the alignment, are colored wheat for the scaffold/trimerization domain, blue for the transport domain, yellow for re-entrant helical hairpin HP1, and red for re-entrant helical hairpin HP2. Dashed green lines represent residues grafted from an extant sequence into ancestral protein constructs in place of poorly aligned regions (see Methods). Filled purple circles mark residues in Glt_Ph_ that coordinate Na^+^ ions with their side chains, filled green squares mark residues in Glt_Ph_ that coordinate L-aspartate with their side chains, and empty circles represent allosteric mutations that can turn Na^+^-coupling on and off. Notable sequence changes in H^+^-coupled transporters compared to Na^+^-coupled transporters are shown in bold, and red residues are changes found at Na^+^-binding sites. (**b**) Structure of a Glt_Ph_ protomer in the outward-facing state (PDB 6UWF), colored as in (**a**). Purple spheres are Na^+^ ions (placed based on PDB 6×15), and the substrate is green.

**Extended Data Fig. 6 |:**
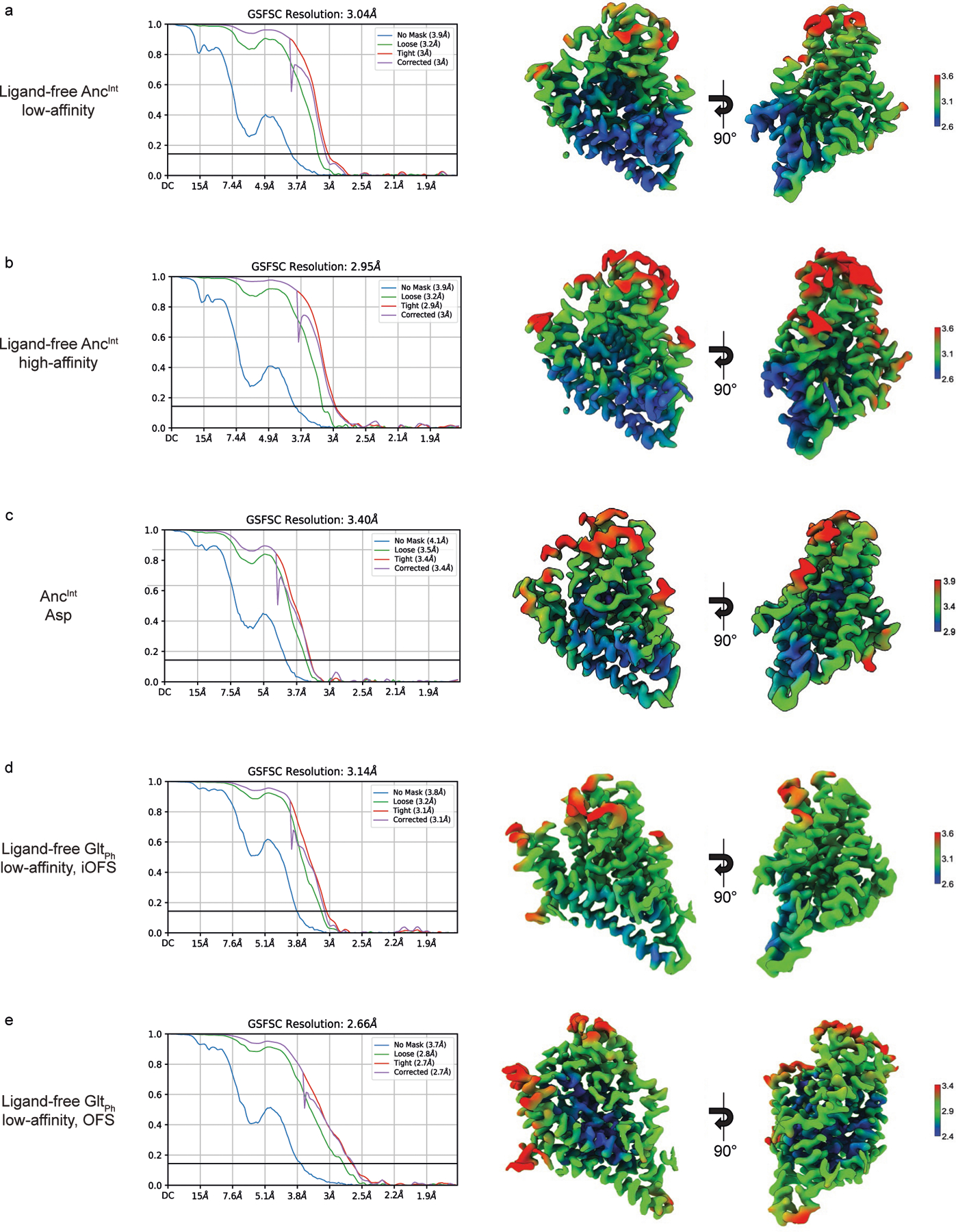
Validation statistics for all structures. Map validations of (**a**) ligand-free Anc^Int^, low-affinity; (**b**) ligand-free Anc^Int^, high-affinity; (**c**) L-aspartate-bound Anc^Int^; (**d**) ligand-free Glt_Ph_, low-affinity, iOFS; (**e**) ligand-free Glt_Ph_, low-affinity, OFS. From left to right, map FSC from local refinement in cryoSPARC and local resolution estimation of the unsharpened map. All maps are contoured at 8 σ. The adjacent protomers (unused in refinement) are removed for clarity.

**Extended Data Fig. 7 |:**
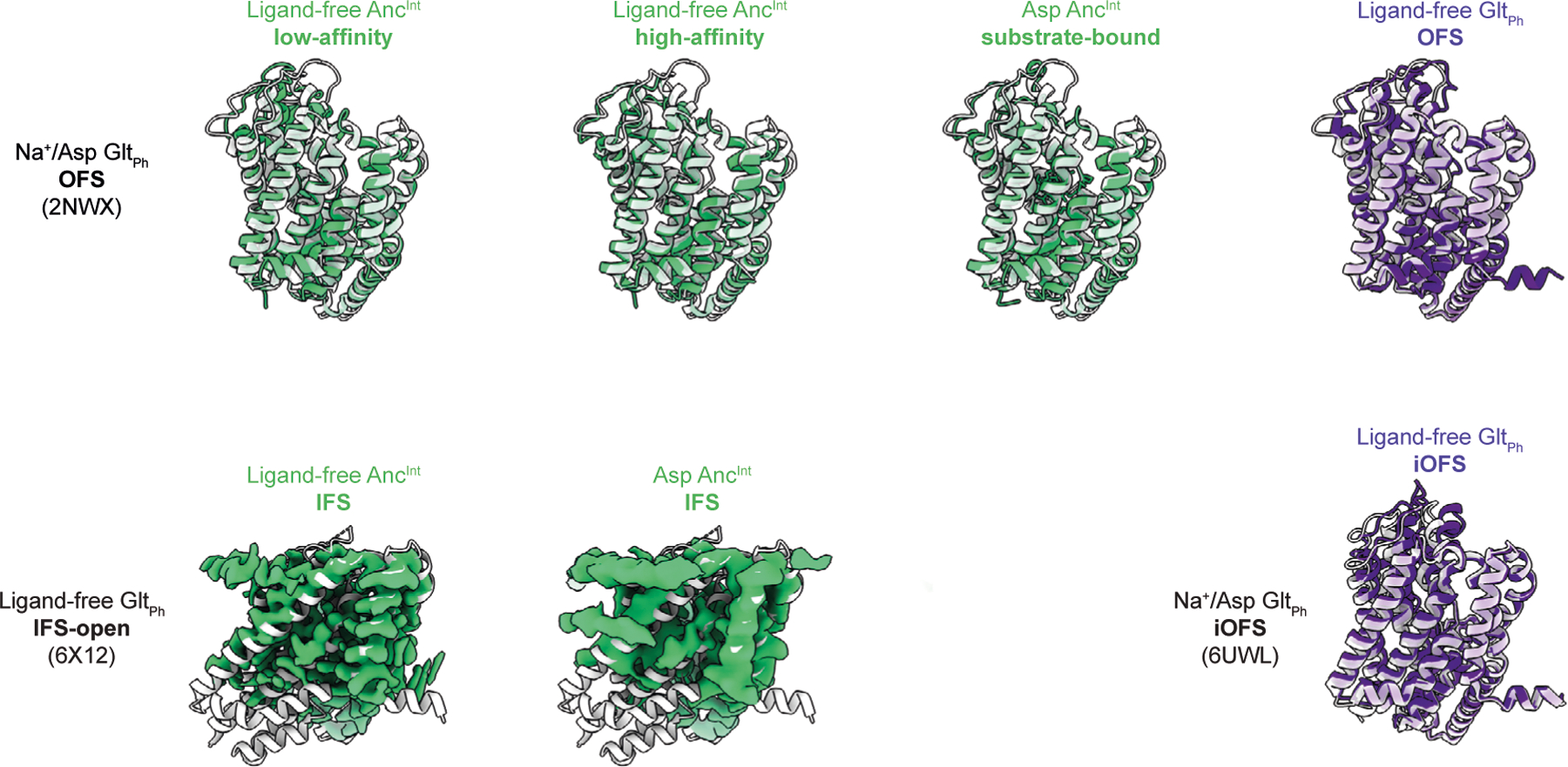
Global transport domain conformations of structures obtained in this study. Anc^Int^ (green) or Glt_Ph_ (purple) structures were aligned on the scaffold domain (residues 1–75, 140–225) of the closest Glt_Ph_ structure in the PDB (translucent white). These Glt_Ph_ structures were either OFS (2NWX) or iOFS (6UWL). IFS states in Anc^Int^ were incompletely resolved, and models could not be built; therefore, the maps from 3D classification were contoured to a σ of 6 and aligned to the closest Glt_Ph_ structure, IFS-open (white, 6 × 12).

**Extended Data Fig. 8 |:**
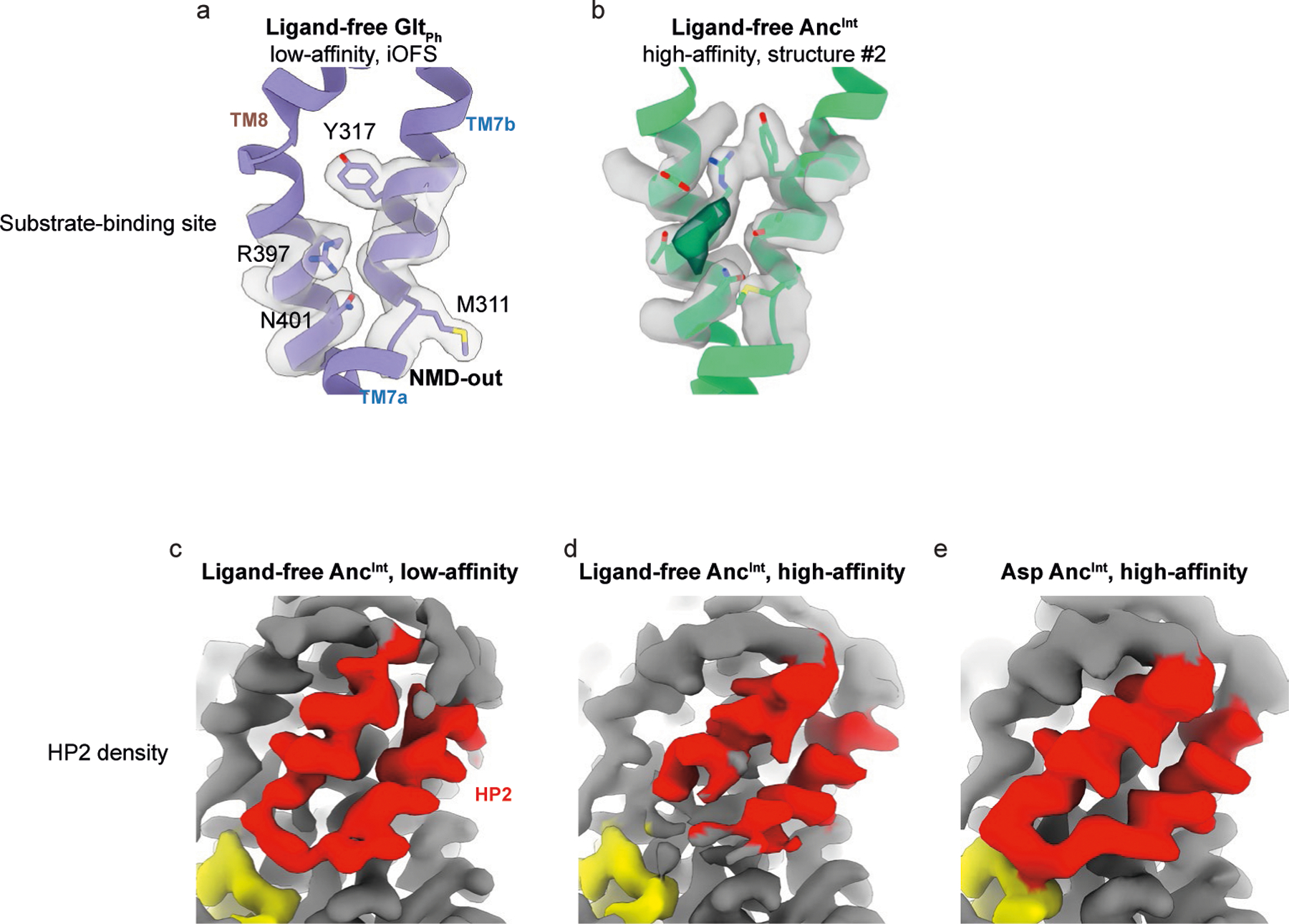
Alternate substrate binding configurations and AncInt HP2 densities. (**a**) Substrate-binding sites of ligand-free iOFS Glt_Ph_ in the low-affinity configuration. (**b**) Substrate-binding site of ligand-free Anc^Int^ in the high-affinity configuration #2. The green density overlaps with the L-aspartate binding site but is too large and differently shaped to correspond to L-aspartate. (**c–e**) HP2 densities of ligand-free Anc^Int^, low-affinity (**c**), ligand-free Anc^Int^, high-affinity (**d**), and L-aspartate Anc^Int^, high-affinity (**e**). Densities are unsharpened and contoured 10 σ. HP1 is in yellow, and HP2 is in red.

**Extended Data Fig. 9 |:**
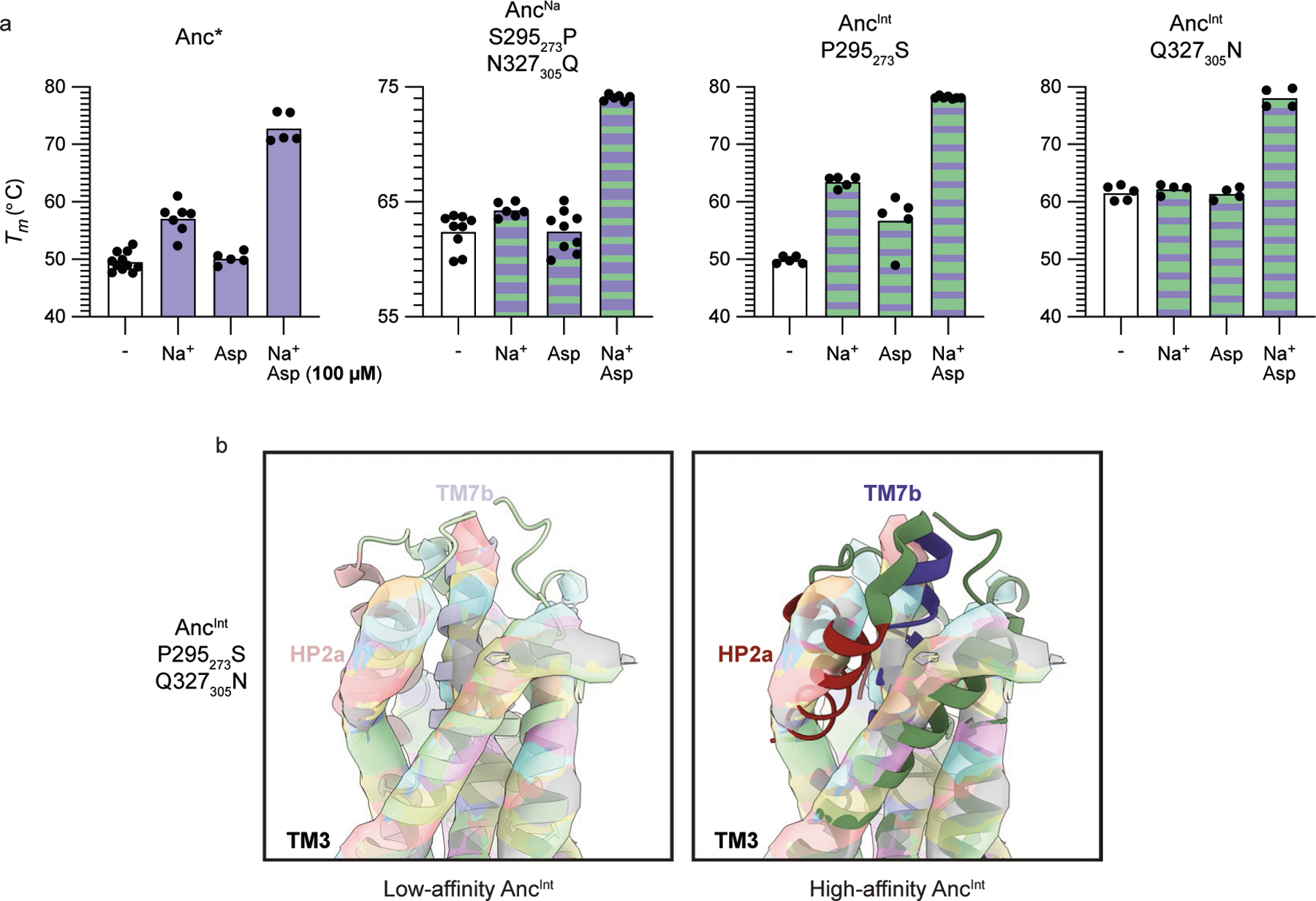
Functional characterization of mutant ancestral proteins. (**a**) nanoDSF assays of Anc*, Anc^Na^ S295_273_P/N327_305_Q, and single Anc^Int^ mutations. Melting temperatures (*T*_*m*_) in buffer alone (ligand-free), Na^+^, L-aspartate, and Na^+^/L-aspartate. Replicates are from at least two independent experiments, each with several technical replicates. All ligand concentrations are 10 mM Na^+^ and 1 mM L-aspartate, unless otherwise indicated. (**b**) Alignment of all 20 classes of Anc^Int^ P295_273_S/Q327_305_N protomers (obtained by 3D classification without alignment, additional detail in Methods) to the scaffold domains of either Anc^Int^ low-affinity or high-affinity states.

**Extended Data Fig. 10 |:**
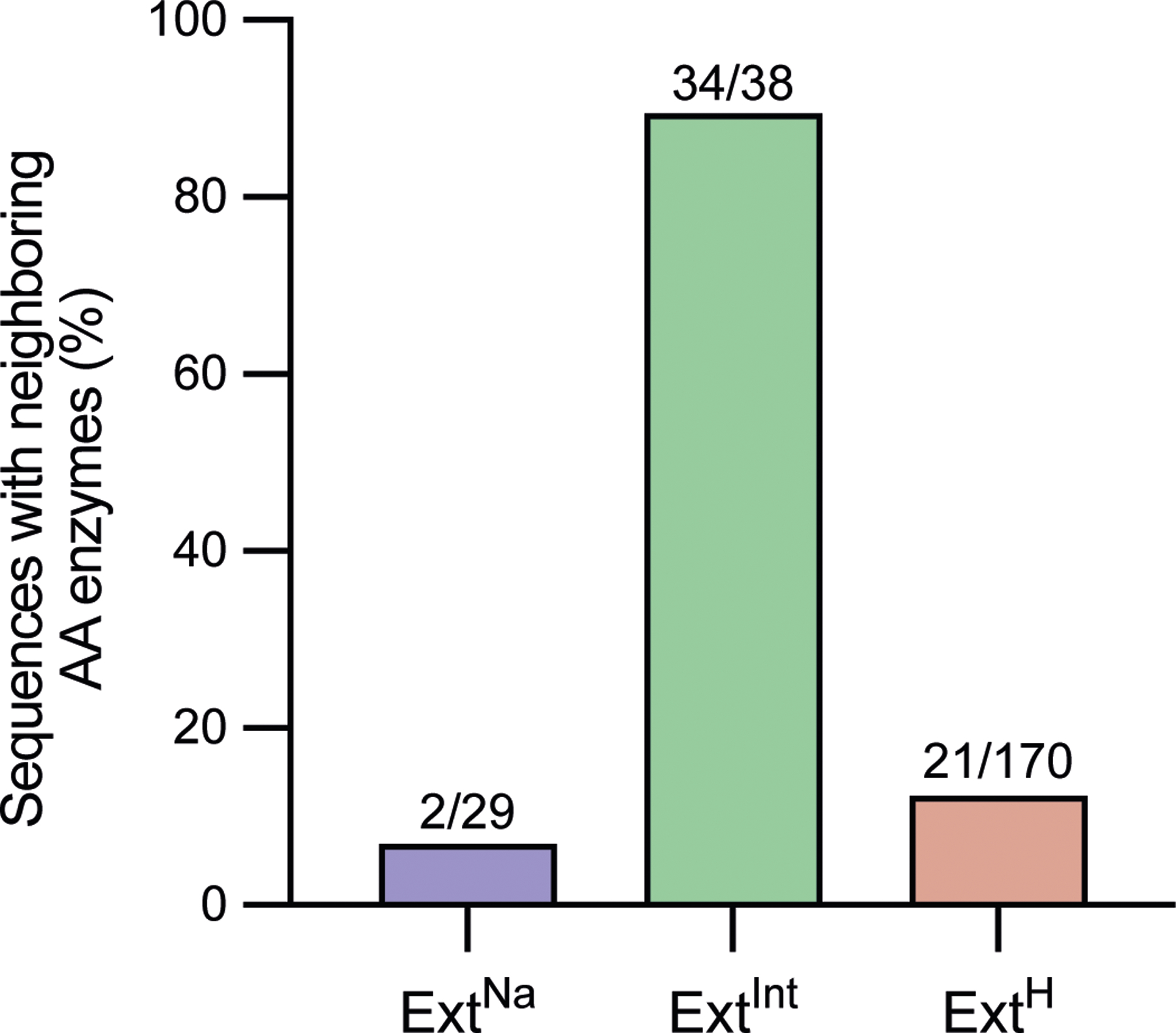
Genomic pairing of uncoupled transporters and enzymes. Transporters were considered to have a paired enzyme if the enzyme was within 3 protein-coding genes away from the transporter and if the enzyme had putative free amino acid/dicarboxylate modifying function according to Pfam annotation. The fraction of sequences with genomic transporter/enzyme pairs is on the top of each bar. Only transporters with available genomic neighborhood information were considered; for the Ext^Na^, Ext^Int^, and Ext^H^ clades, this corresponds to 29/186, 38/161, and 170/786, respectively.

## Supplementary Material

Supplementary Information

Supplementary Data 2

The online version contains supplementary material available at https://doi.org/10.1038/s41594-025-01652-z.

## Figures and Tables

**Table 1 |:** Cryo-EM data collection, refinement and validation statistics

	Anc^Int^, ligand-free, low affinity (EMD-44526) (PDB 9BGY)	Anc^Int^, ligand-free, high affinity (EMD-44527) (PDB 9BGZ)	Anc^Int^, l-aspartate-bound (EMD-44528) (PDB 9BH0)	Glt_Ph_, ligand-free, intermediate outward-facing (EMD-44529) (PDB 9BH1)	Glt_Ph_, ligand-free, outward-facing (EMD-44530) (PDB 9BH2)
**Data collection and processing**					
Magnification	×105,000	×105,000	×105,000	×81,000	×81,000
Voltage (kV)	300	300	300	300	300
Electron exposure (e^−^ per Å^2^)	50.29	50.29	58.00	53.42	53.42
Defocus range (μm)	−0.9 to −2.1	−0.9 to −2.1	−0.9 to −2.1	−0.9 to −2.1	−0.9 to −2.1
Pixel size (Å)	0.4125	0.4125	0.4165	0.4230	0.4230
Symmetry imposed	*C*_1_	*C*_1_	*C*_1_	*C*_1_	*C*_1_
Initial particle images (no.)	3,543,864	3,543,864	954,856	1,682,016	1,682,016
Final particle images (no.)	68,420	63,869	71,731	157,756	148,756
Map resolution (Å), masked FSC= 0.143	3.0	3.0	3.4	3.1	2.7
Map resolution range (Å)	2.6–4.4	2.6–4.7	2.9–5.6	2.7–4.8	2.4–4.6
**Refinement**					
Initial model used (PDB code)	2NWX (SWISS-MODEL)	2NWX (SWISS-MODEL)	2NWX (SWISS-MODEL)	6UWL	6UWF
Model resolution (Å)	3.2/2.9	3.2/3.0	3.6/3.3	3.4/3.1	3.3/2.7
FSC= 0.5/0.143					
Model resolution range (Å)					
Map sharpening *B* factor (Å^2^)	−81.6	−76.4	−96.5	−93.3	−57.7
Model composition					
Nonhydrogen atoms	2,916	2,783	2,935	2,942	3,046
Protein residues	389	369	391	395	414
Ligands	0	0	1	0	0
*B* factors (Å^2^), mean					
Protein	95.23	72.77	104.81	101.18	87.00
Ligand			111.09		
Root-mean-square deviations					
Bond lengths (Å)	0.002	0.003	0.002	0.003	0.003
Bond angles (°)	0.499	0.542	0.573	0.482	0.523
Validation					
MolProbity score	1.36	1.42	1.26	1.30	1.37
Clashscore	6.28	7.60	4.94	5.60	6.79
Poor rotamers (%)	0.32	0.66	0.32	0.00	0.62
Ramachandran plot					
Favored (%)	97.93	98.35	98.20	99.74	98.30
Allowed (%)	2.07	1.65	1.80	0.26	1.70
Disallowed (%)	0.00	0.00	0.00	0.00	0.00

FSC, Fourier shell correlation.

## Data Availability

Atomic coordinates and EM densities were deposited to the PDB and EM Data Bank for the following structures: ligand-free Anc^Int^ low-affinity (PDB 9BGY, EMD-44526); ligand-free Anc^Int^ high-affinity (PDB 9BGZ, EMD-44527); l-aspartate-bound Anc^Int^ (PDB 9BH0, EMD-44528); ligand-free Glt_Ph_ intermediate outward-facing (PDB 9BH1, EMD-44529); ligand-free Glt_Ph_ outward-facing (PDB 9BH2, EMD-44530). Supplementary Data 2 includes all input and output data related to phylogenetic analyses, plasmid maps of all protein expression constructs used in this study and a ChimeraX session file containing comparison of Anc^Int^ models to Anc^Int^ P295_273_S;Q327_305_N maps (Extended Data Figure 9). Plasmid backbones designed in this study are available on Addgene (pBAD24-ALFA, 240248; pBAD24-TGP, 240249; pET15b-ALFA, 240252; pET15b-TGP, 240251). Previously published structural models are available from the PDB under accession codes 2NWX, 6UWF, 6X12, 6X15, 7AHK, 7RCP, 8CUI and 8CV2. All other plasmids and data are available upon request. Source data are provided with this paper.
