# Supplementary material for: Evolutionary analysis reveals the origin of sodium coupling in glutamate transporters
Source: Nat Struct Mol Biol. Author manuscript; Available in PMC 2025 Dec 2. (PMC12668577; doi:10.1038/s41594-025-01652-z)
Supplement: Supplementary Information [file NIHMS2118805-supplement-Supplementary_Information.pdf]

nature structural & molecular biology

Supplementary information https://doi.org/10.1038/s41594-025-01652-z

Evolutionary analysis reveals the origin of sodium coupling in glutamate transporters

In the format provided by the authors and unedited

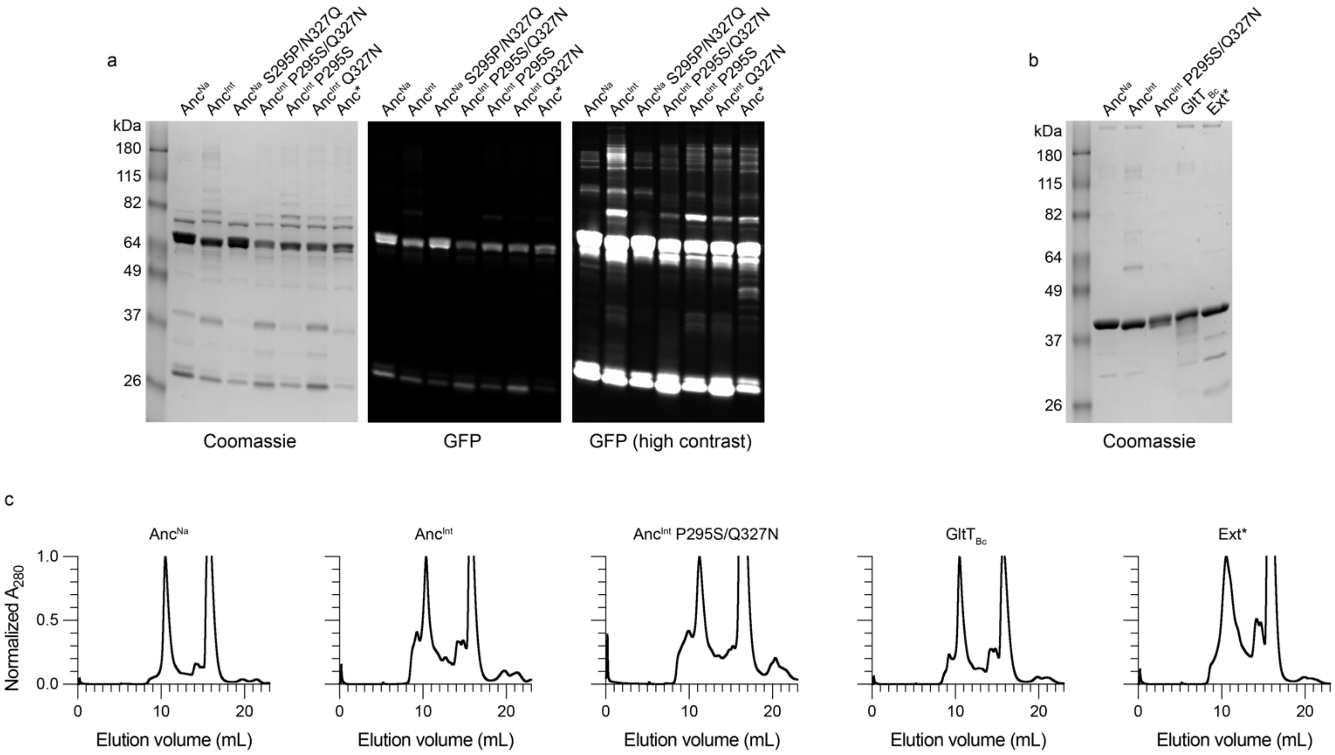

Supplementary Figure 1. Representative gels and size-exclusion chromatography profiles of all proteins used in this study. All residue numbering corresponds to Glt_{Ph} numbering. (a) 12% Tris-Glycine gels (Coomassie, GFP) of protein used in nanoDSF experiments, purified via Streptactin columns (described in Methods). Estimated 1.5 µg protein loaded per lane. Most Coomassie bands correspond to high-contrast GFP bands, suggesting high purity. (b) 12% Tris-Glycine Coomassie gel of size-exclusion purified GFP-free protein used for proteoliposome experiments. Protein was purified via Streptactin columns, cleaved with thrombin, and subsequently further purified via size-exclusion chromatography (described in Methods). Estimated 1.5 µg protein loaded per lane. (c) Representative size-exclusion profiles (Superdex 200 Increase, 10/300) of purified protein corresponding to panel (b), normalized to main protein peaks ~10.5-11.3 mL. Large peaks outside the y-axis limits (~16.5 mL) are cleaved GFP.

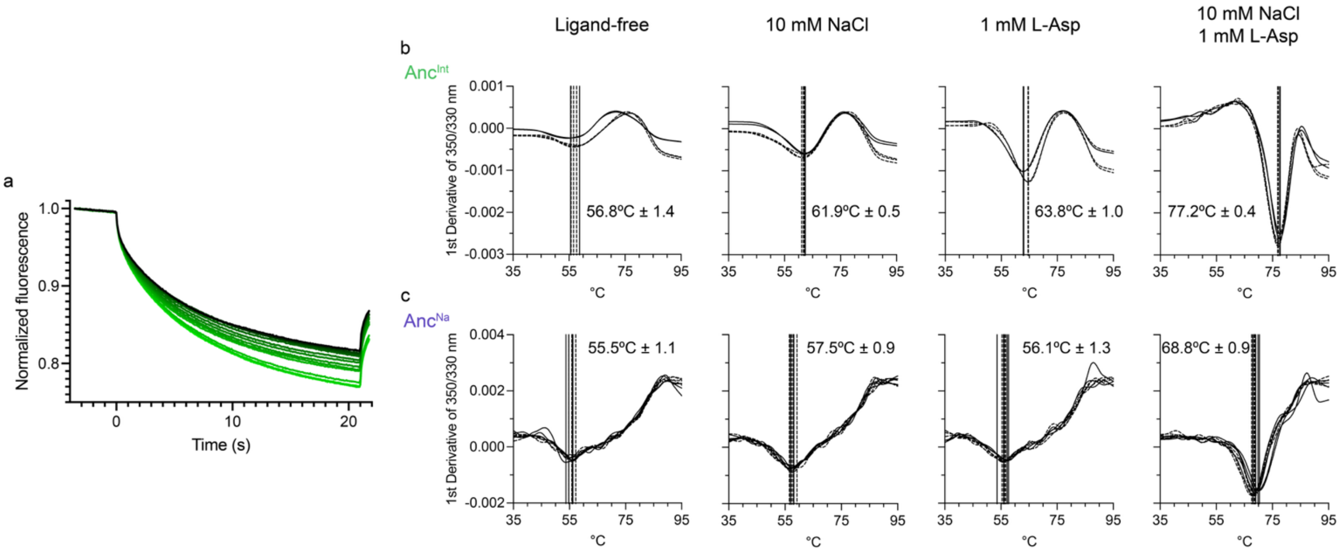

Supplementary Figure 2. L-Asp and Na^+ binding to Anc^{Na} and Anc^{Int}. (a) Representative raw MST traces from an individual binding affinity experiment. Traces represent the MST signals of GFP-tagged Anc^{Int} in the presence of 100 mM Na^+ at various L-Asp concentrations. Lighter green lines correspond to increasing L-Asp concentrations. Fraction-bound values in the main text are calculated between 1.5-5s response time, depending on the individual experiment. (b-c) Raw first-derivative curves of data displayed in Figure 3, to determine thermostability as measured by nanoDSF of Anc^{Int} (b) and Anc^{Na} (c). Different pattern lines represent traces performed on independent biological replicates. Vertical lines represent T_m-s estimated for individual curves. Mean values with standard deviations are shown on the panels.

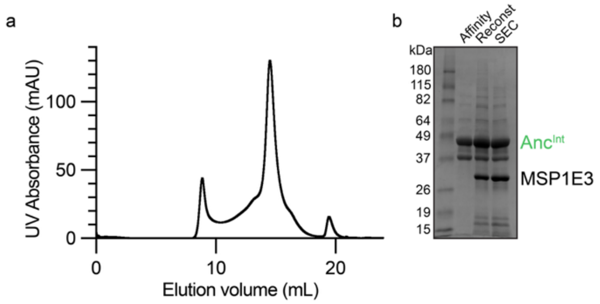

Supplementary Figure 3. Nanodisc reconstitution of Anc^{Int}. (a) Representative size-exclusion chromatography profile of GFP-free Anc^{Int} reconstituted into MSP1E3 nanodiscs. The column is a Superose 6 Increase 10/300 GL, and the buffer is 20 mM HEPES pH 7.4, 100 mM NMDG-Cl. (b) Representative Coomassie-stained SDS PAGE of GFP-free Anc^{Int} and reconstitution into MSP1E3 nanodiscs. “Affinity” refers to pooled and concentrated elution fractions following Streptactin XT affinity purification. “Reconst” refers to Anc^{Int} successfully reconstituted into MSP1E3 nanodiscs after BioBead application and ultracentrifugation. “SEC” refers to pooled and concentrated peak fractions of Anc^{Int}/MSP1E3 eluting at ~14.5 mL following size-exclusion chromatography.

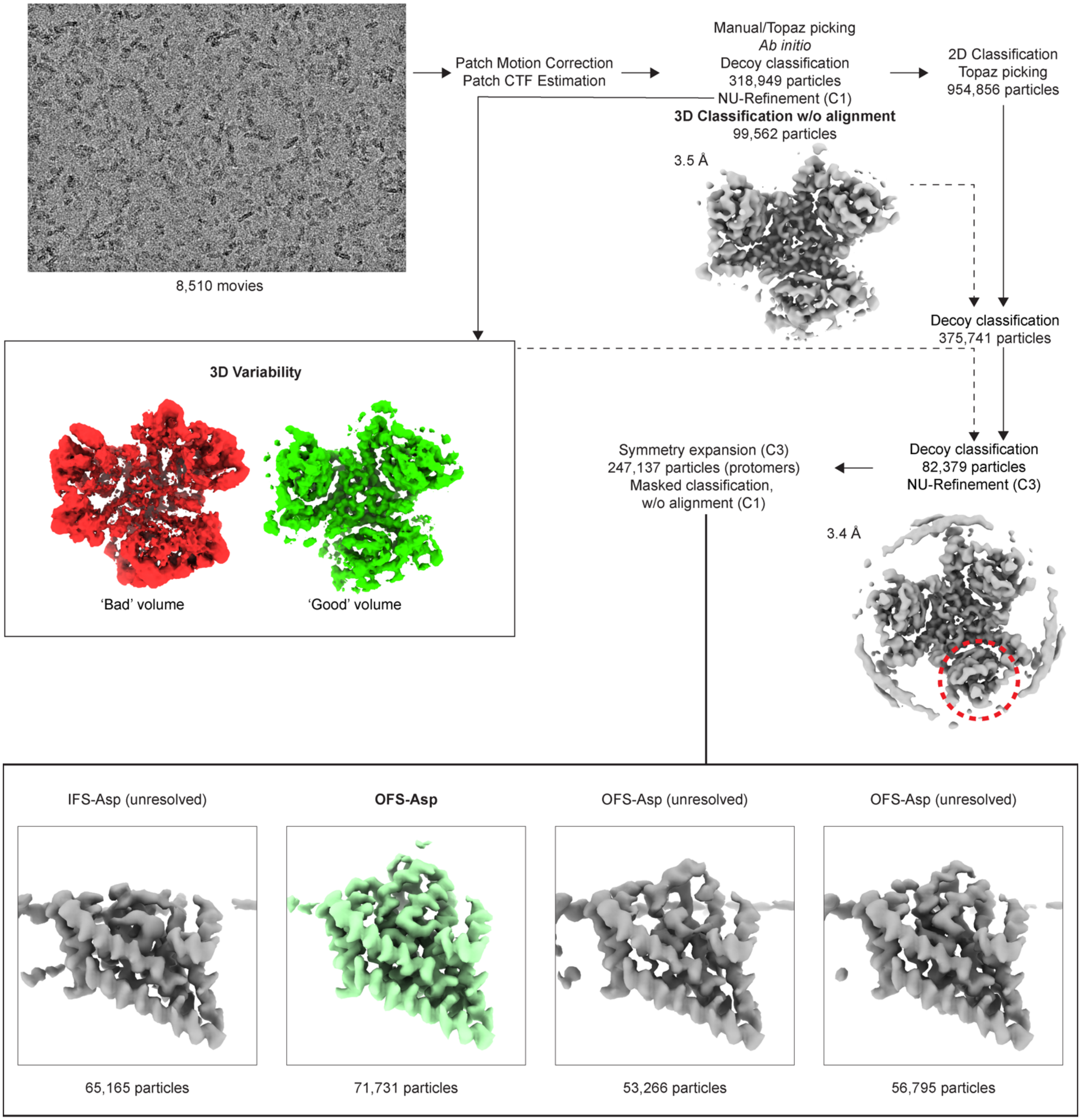

Supplementary Figure 4. Processing workflow of Anc^{Int}, substrate-bound conditions. Steps are described in further detail in Methods. All steps were performed in cryoSPARC. 'Decoy classification' is a nickname for heterogeneous refinement using 'decoy' noise volumes and a 'good' and 'bad' volumes obtained from 3D Variability (Methods). The dashed red circle is the approximate location of the mask used for masked 3D classification. All maps are unsharpened and contoured to a σ of 10. Colored protomers were used for further local refinement and model building, with the other two protomers removed for clarity.

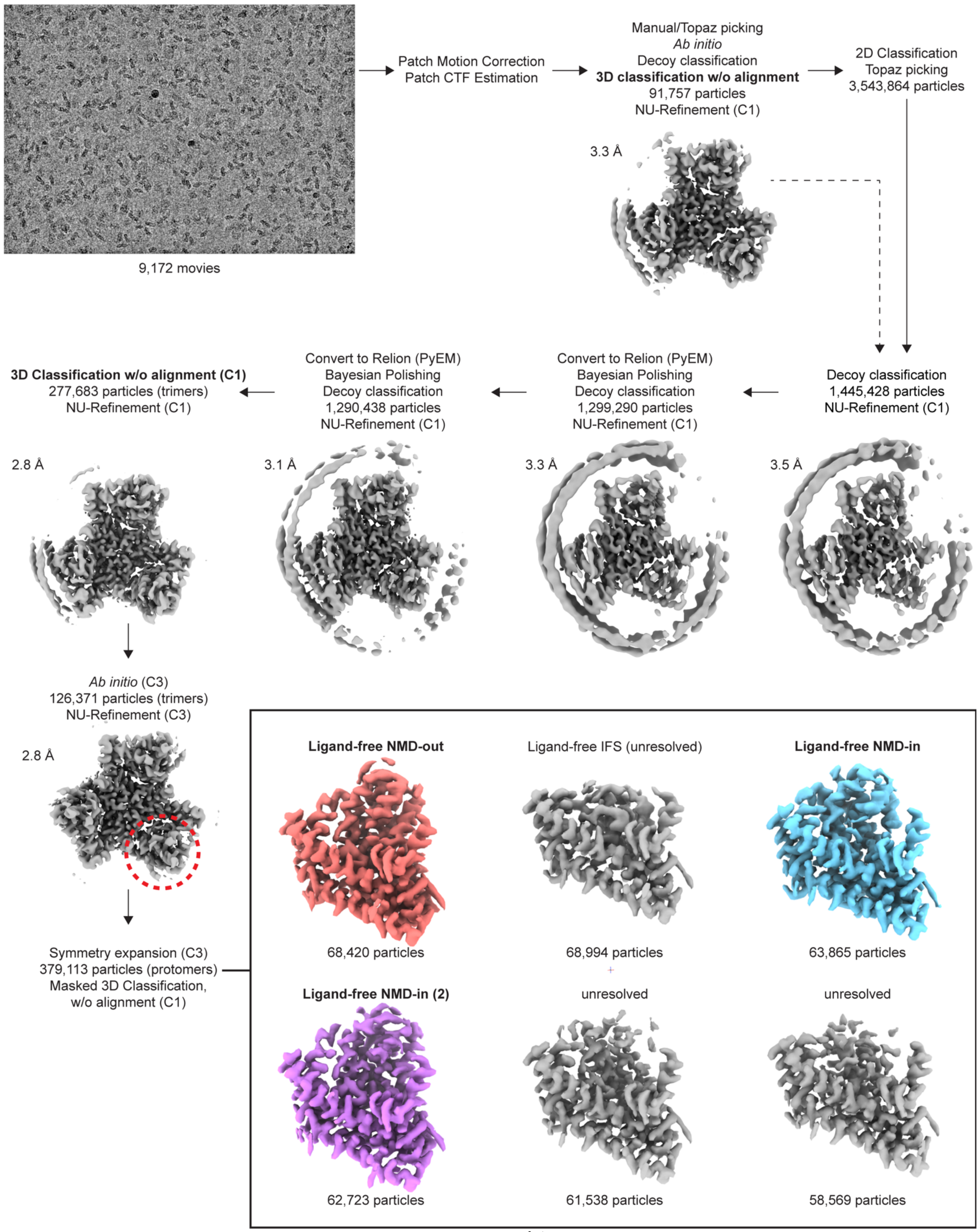

Supplementary Figure 5. Processing workflow of Anc^{Int}, ligand-free conditions. Steps are described in further detail in Methods. All steps were performed in cryoSPARC except for particle stack conversion to Relion (PyEM) and Bayesian Polishing (Relion). 'Decoy classification' is a nickname for heterogeneous refinement using 'decoy' noise volumes (Methods). The dashed red circle is the approximate location of the mask used for masked 3D classification. All maps are unsharpened and contoured to a σ of 10. Colored protomers were used for further local refinement and model building, with the adjacent two protomers removed for clarity.

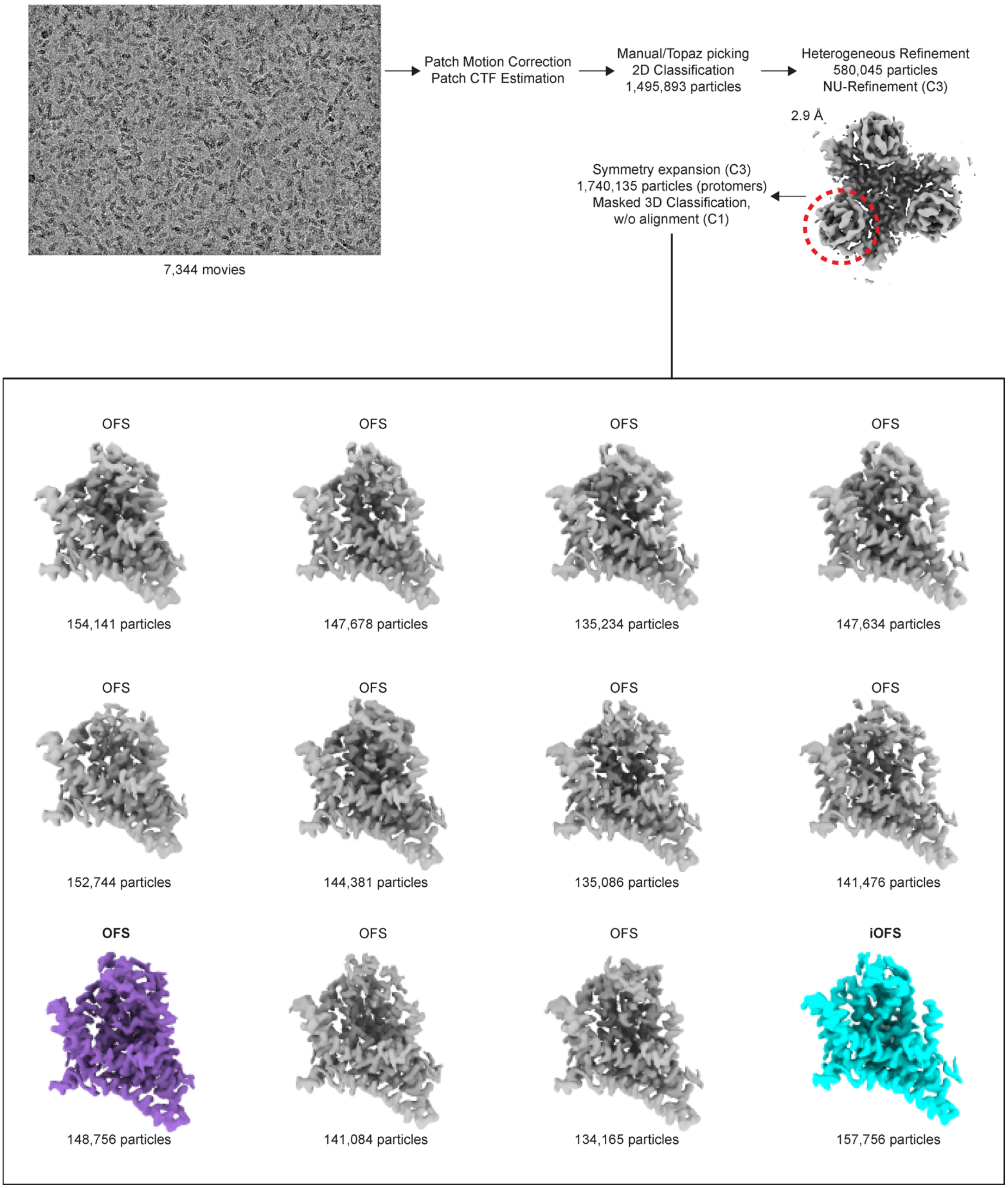

Supplementary Figure 6. Processing workflow of Glt_{Ph}, ligand-free conditions. Steps are described in further detail in Methods. All steps were performed in cryoSPARC. The dashed red circle is the approximate location of the mask used for masked 3D classification. All maps are unsharpened and contoured to a σ of 10. Colored protomers were used for further local refinement and model building, with the other two protomers removed for clarity.

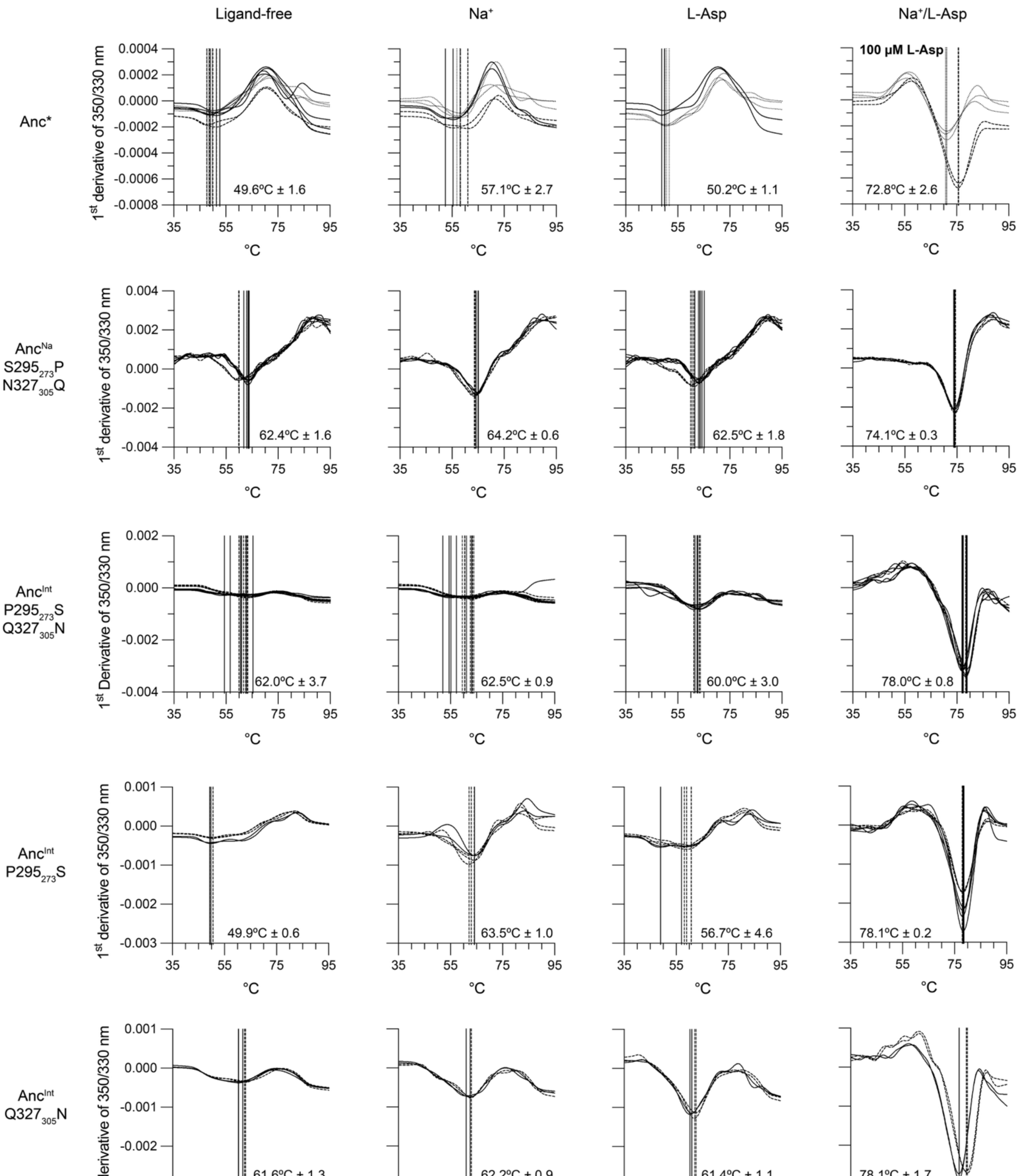

Supplementary Figure 7. Raw nanoDSF first-derivative curves of Anc*, Anc^{Na} S295$_{273}$P/N327$_{305}$Q, Anc^{Int} P295$_{273}$S/Q327$_{305}$N, and single Anc^{Int} mutations (P295$_{273}$S or Q327$_{305}$N). Melting temperatures (T_m) were measured in buffer alone (ligand-free), Na^+, L-Asp, and Na^+/L-Asp. All ligand concentrations are 10 mM Na^+ and 1 mM L-Asp, unless otherwise indicated. Replicates are from at least two independent experiments. Different pattern lines represent traces performed on independent biological replicates. Vertical lines represent T_m-s estimated for individual curves. Mean values are shown on the panels.

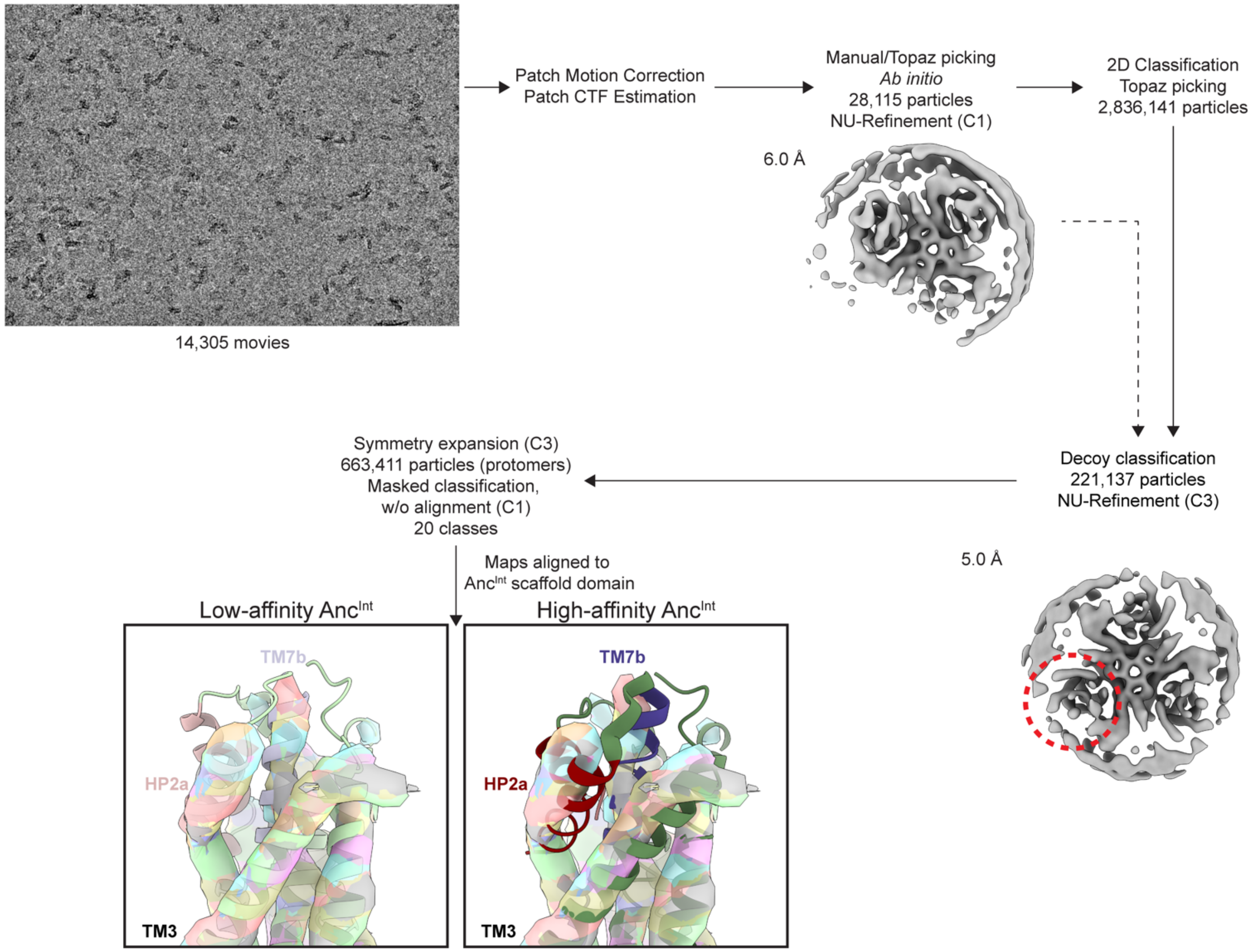

Supplementary Figure 8. Processing workflow of Anc^{Int} P295$_{273}$S/Q327$_{305}$N, ligand-free conditions. All steps were performed in cryoSPARC. The dashed red circle is the approximate location of the mask used for 3D classification without alignment, following symmetry expansion. All maps are unsharpened and contoured to a σ of 10. The scaffold domains of Anc^{Int} low-affinity or high-affinity states are aligned to all 20 classes of protomers obtained by 3D classification without alignment.

	Glt_{Ph}	EAAT3	Anc^{Int}
**Low-affinity PDB ID**	9BH2	8CUI	9BGY
**High-affinity PDB ID**	7AHK	8CV2	9BGZ
**Average TM7b Cα RMSD (Å)**	0.45	0.69	0.37
**First residue Cα deviations (Å)**	0.78	1.04	0.74
**Last residue Cα deviations (Å)**	2.54	1.72	0.95
**Cα rotation angle (°)**	4.9	8.0	2.1
**Shift along axis (Å)**	-0.62	-0.70	-0.03

Supplementary Table 1: Comparison of TM7b in low- and high-affinity states. Structures were aligned on HP1 (residues: Glt_{Ph} 257-292, EAAT3 312-347, Anc^{Int} 236-270) using the ChimeraX matchmaker tool; per-residue Cα RMSD was concurrently calculated. Average RMSD, rotation angle, and shift along axis were calculated at Cα atoms of TM7b (residues: Glt_{Ph} 315-329, EAAT3 371-385, Anc^{Int} 293-307) using the reportMatrix option in the ChimeraX align tool.
